# Light- and circadian-controlled genes respond to a broad light spectrum in Puffer Fish-derived Fugu eye cells

**DOI:** 10.1038/srep46150

**Published:** 2017-04-18

**Authors:** Keiko Okano, Shoichi Ozawa, Hayao Sato, Sawa Kodachi, Masaharu Ito, Toshiaki Miyadai, Akihiro Takemura, Toshiyuki Okano

**Affiliations:** 1 Department of Electrical Engineering and Bioscience, Graduate School of Advanced Science and Engineering, Waseda University, Wakamatsu-cho 2-2, Shinjuku-ku, Tokyo 162-8480, Japan; 2 Department of Marine Bioscience, Faculty of Marine Bioscience, Fukui Prefectural University, 1-1 Gakuen-cho Obama, Fukui 917-0003, Japan; 3 Department of Chemistry, Biology, and Marine Science, Faculty of Science, University of the Ryukyus, 1 Senbaru, Nishihara-cho, Nakagami-gun, Okinawa 903-0213, Japan

## Abstract

Some cell lines retain intrinsic phototransduction pathways to control the expression of light-regulated genes such as the circadian clock gene. Here we investigated the photosensitivity of a Fugu eye, a cell line established from the eye of *Takifugu rubripes*, to examine whether such a photosensitive nature is present. Microarray analysis identified 15 genes that showed blue light-dependent change at the transcript level. We investigated temporal profiles of the light-induced genes, as well as *Cry* and *Per,* under light-dark, constant light (LL), and constant dark (DD) conditions by quantitative RT-PCR. Transcript levels of *Per1a* and *Per3* genes showed circadian rhythmic changes under both LL and DD conditions, while those of *Cry* genes were controlled by light. All genes examined, including DNA-damage response genes and photolyase genes, were upregulated not only by blue light but also green and red light, implying the contribution of multiple photopigments. The present study is the first to identify a photosensitive clock cell line originating from a marine fish. These findings may help to characterize the molecular mechanisms underlying photic synchronization of the physiological states of fishes to not only daily light-dark cycles but also to various marine environmental cycles such as the lunar or semi-lunar cycle.

Light greatly impacts certain physiological responses in living organisms through the activation of intrinsic photopigments such as opsins. Light-activated opsins can trigger intracellular transduction pathways to generate electrical responses in visual photoreception, while light-activated gene transcription can trigger growth responses such as plant photomorphogenesis. In animals, light-induced changes in mRNA levels have been studied in connection with daily or circadian rhythms^1^, photorepair of UV-damaged DNA^2^, and photoperiodic changes in seasonal response^3^.

The circadian clock oscillates with a period of approximately 24 h and is essential to synchronizing daily behavior and homeostasis with light and dark changes in the external environment. The basic structure of animal circadian clock oscillators contains feedback loops based on the transcription and translation^4^,^5^. Within the central mechanism of the oscillator, a limited number of transcription factors activate or inhibit cis elements and display peak transcriptional activity at different times of the day^6^. The E/E’-box seems to be the most important of the cis elements for oscillatory function, and it is a target of positive elements BMAL and CLOCK, to which negative factors CRY and PERIOD bind to inhibit the transactivation activity of many clock-controlled genes including *Per* and *Cry* genes themselves. The circadian oscillation system is mostly present in individual animal cells even in the peripheral tissues. In animals other than mammals, the cellular oscillator is closely linked with an endogenous light-input pathway for synchronization of the clock to external light-dark cycles^5^,^7^.

In the phase-shift mechanism of the fruit fly circadian clock, cryptochrome (dCRY) uses environmental light to trigger its own degradation or that of dTIM^8^, while in vertebrates the induction of clock genes such as *Per*^9^ or *E4bp4*^10^ may be important for phase shift of the circadian clock. In order to elucidate the phase-shift mechanisms involved in the vertebrate circadian clock, photosensitive clock cells have been the focus of studies. The chick pineal gland and primary cultured pineal cells contain photosensitive clock genes that have been used to study the phototransduction cascade of the circadian oscillator^11^.

Visible light-dependent induction of gene expression was shown in RBCR-1 and OL32 cell lines, which were derived from the caudal fin of a goldfish and medaka, respectively^12^,^13^. In these cell lines, CPD-photolyase (*CPD-Phr*), a gene encoding a photorepair enzyme, responded to fluorescent light, but there was no information about the induction mechanism or the presence of a circadian clock. In 2000, the zebrafish cell line PAC-2 was reported to contain a photosensitive circadian clock^7^. Thereafter, zebrafish photosensitive clock cell lines have been used as photosensitive clock models instead of the primary chick pineal cells.

Although many studies using the fish photosensitive cells focus on circadian photopigments and the photic input pathway^14^,^15^,^16^,^17^,^18^,^19^,^20^,^21^,^22^,^23^,^24^,^25^,^26^, the specificity and generality of the photic gene regulation mechanism in lower vertebrates remains unclear. This is partly due to the high differentiation of systems within a single species. For example, even among the zebrafish photosensitive clock cell lines, the z*Per2* gene is induced by blue light but not red light in Z3 cells^15^, while it is induced by both blue and red light in PAC-2 cells^25^. Another reason for the lack of clarity may be the concentration of studies on zebrafish cells and the lack of a model cell line that originates from different species. In particular, the teleost species have a diverging repertoire of circadian clock genes due to lineage-specific gene duplications and differential gene deletions^27^. Therefore, we considered the search for another suitable fish cell line important for comparative analyses of the photoresponses and clock mechanisms across species.

In this study, we found the Fugu eye cell line to be a model cell line that is ideal for this purpose. Fugu eye cells are derived from *Takifugu rubripes (Fugu rubripes*)^28^, a species with the most compact genome retaining a gene repertoire similar to that in the human genome^29^. Its size (haploid) of 400 Mb is only 11% and 25% the size of humans and zebrafish, respectively[www.genomesize.com], and its repetitive sequences are less than 10%^30^. These virtues offer several advantages in the comparative genomics, especially in their inference of possible gene regulatory elements. *T. rubripes* is also one of the most expensive aquaculture resources in Japan, and is likely important in that it is not only ecologically but also evolutionarily closer to many edible marine fish species in the *Acanthopterygii* superorder than the zebrafish. We searched for light-induced or clock-controlled genes using both microarray analysis and qRT-PCR. Fifteen genes were identified, including the clock (-related) genes, and were further classified into two groups on the basis of their temporal expression profiles. *TrPer1a* and *TrPer3* showed a clear oscillation under both constant light and dark conditions. On the other hand, mRNA levels of *TrCry* and photolyase genes, except for *TrCry3*, were exclusively controlled by light. These genes were induced by not only blue light but also green and red light, indicating that multiple photopigments or a novel photopigment with a broad spectral range likely contribute to the light-dependent gene regulation in *T. rubripes*.

## Results

### Microarray analysis of gene expression in light-exposed Fugu eye cells

**Figure 1:**
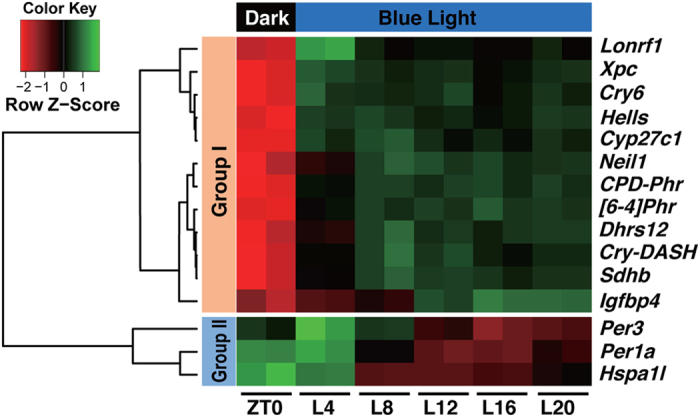
Microarray analysis of temporal changes upon blue light irradiation of Fugu eye cells. Fugu eye cells were entrained to blue light/dark cycles for 4 days and maintained under blue light for 20 h. The cells were harvested every four hours and subjected to microarray analysis. Data from duplicate arrays for each time point were combined. Fifteen genes were considered to show significant temporal changes. The dendrogram shows relationships between expression profiles. Intensity of blue light: 100 μW/cm^2^ (3.9 μmol photons/s/m^2^).

In our preliminary experiments, we surveyed vertebrate cell-lines and found that the expression of clock genes is upregulated by blue light in Fugu eye cells. Then, we aimed to identify light-responsive genes in this cell line by microarray analysis. Fugu eye cells were entrained to 12 h light (blue)/12 h dark cycles and collected before (ZT0) and at 4, 8, 12, 16 and 20 h after blue light exposure (Fig. 1; L4, L8, L12, L16, and L20). The total RNA samples from the Fugu eye cells were subjected to microarray analysis, from which 15 genes showed significant change (p < 0.01)(Fig. 1, Table 1).

**Figure 2:**
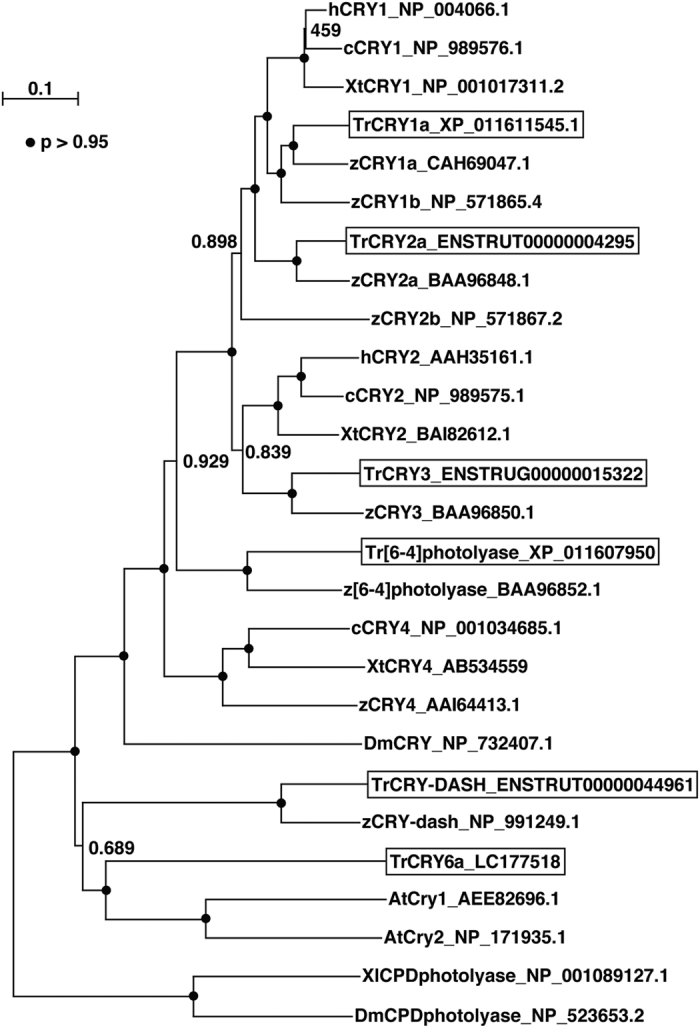
A phylogenetic tree of CRY family proteins constructed by Neighbour-Joining method. The tree is constructed by ClustalW2.1 (http://clustalw.ddbj.nig.ac.jp/) with default parameters. Bootstrap probabilities (P) are symbolized by closed circles on the nodes (P > 95%) or by values near the nodes. h, human; c, chicken; Xt, *Xenopus tropicalis*; Tr, *Takifugu rubripes*; z, zebrafish; Xl, *Xenopus laevis*; Dm, *Drosophila melanogaster*; At, *Arabidopsis thaliana*. Accession numbers are shown after the protein names.

The expression profiles of 15 genes were further clustered into 2 groups (Fig. 1, Table 1) by Bayesian inference based on the cosine distances among the vectors. In total, 12 genes were classified into Group I. Although genes in this group could be further divided into two subclusters in the dendrogram in Fig. 1, there was no clear subcluster-dependent difference in the light responsiveness (see below). In Group I, we found a gene encoding a cryptochrome-like novel protein (tentatively termed *Takifugu rubripes* CRY6[TrCRY6]). We isolated the cDNA from the entire coding sequence of TrCRY6 and identified the two putative polymorphic variants, which showed greater than 99% amino acid identity, as *Takifugu rubripes* CRY6a[TrCRY6a, LC177518] and CRY6b[TrCRY6b, LC177519]. Because the primers for *TrCry6* (Table 1) were exactly matched to both of the variants, we refer to them as *TrCry6* below. TrCRY6 is more closely related to plant CRYs than to any other CRYs found in animals (Figs 2 and S1). Genes encoding proteins involved in DNA-damage responses (TrXPC, TrHELLS, and TrNeil1) and photorepair enzymes (TrCPD-Phr, Tr[6-4]Phr, and TrCRY-DASH) were identified in Group I. Two *Per* genes (*TrPer3* and *TrPer1a*) and *TrHspa1l* showed biphasic temporal changes during constant blue light exposure and were classified into Group II.

### Quantitative RT-PCR analysis of light-induced gene transcripts in Fugu eye cells

**Figure 3:**
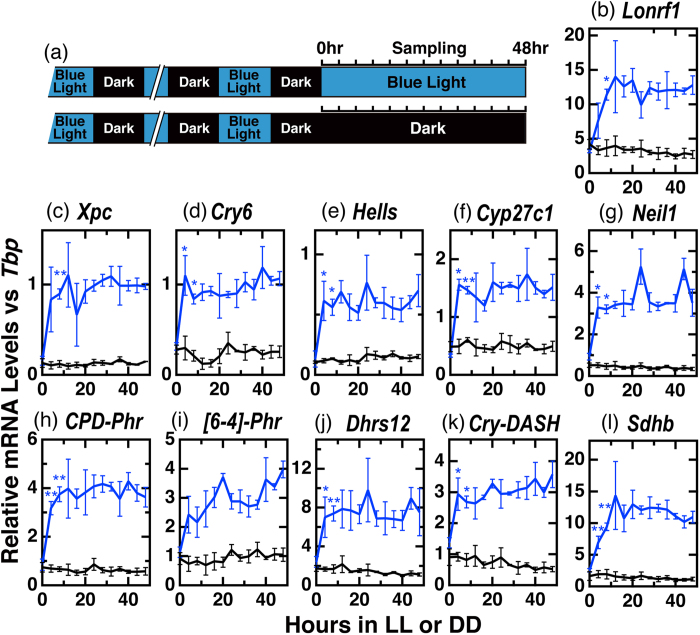
Temporal profiles of mRNA levels of Group I genes by qRT-PCR analysis. (**a**) Schematic representation of the experimental design. Fugu eye cells in culture dishes (n = 3) were entrained to blue light (100 μW/cm^2^)/dark cycles for 4 days and maintained under LL/DD conditions for 48 h. (**b–l**) Quantitative RT-PCR analyses (qRT-PCR) of mRNA levels of Group I genes in Fugu eye cells under constant blue-light (LL, blue lines) or dark (DD, black lines) conditions. Intensity of blue light: 100 μW/cm^2^ (3.9 μmol photons/s/m^2^). In each panel, points are plotted as the means of three independent experiments +/−SE. Asterisks denote significant differences (*q < 0.05; **q < 0.01) between light and dark points, where q-values are p-values adjusted for multiple comparisons at two time points (4 h and 8 h) using the BH method (Table 2).

Through quantitative RT-PCR analyses, we further investigated temporal changes in the mRNA expression levels of Group I genes. Light exposure was extended for 48 h in order to examine the temporal nature of the light-dependent regulatory responses (Fig. 3, Table S1). Although we did not detect amplification of *TrIgfbp4* cDNA, all of the other Group I genes showed significant light-dependent upregulation, and their profiles were substantially similar to each other (Fig. 3). The microarray analysis (Fig. 1) showed that expression levels of Group I genes seemed to be acutely upregulated within 8 h and reached near constant levels after 8 h of irradiation. We then carried out statistical analyses of the effect of light irradiation for 4 h, 8 h, and 8–48 h of light irradiation separately (Tables 2 and 3). Eight genes in Group I, *TrCry6, TrHells, TrCyp27c1, TrNeil1, TrCPD-phr, TrDhrs12, TrCry-dash*, and *TrSdhb*, significantly responded to light within 4 h (Table 2, p < 0.05, q < 0.05), and the other 3 genes, *TrLonrf1, TrXpc*, and *Tr[6-4]Phr*, likely responded to light within 8 h (Table 2, p < 0.05, q ≤ 0.0525). The mRNA levels of all the 11 examined genes (Fig. 3) are significantly higher in the light-irradiated cells than the dark-incubated cells after 8 h light treatment, although significant interactions between the light conditions and time were observed in *Tr[6-4]Phr* and *TrCry-DASH* (Table 3).

### Quantitative RT-PCR analysis of clock (-related) gene transcripts in Fugu eye cells

**Figure 4:**
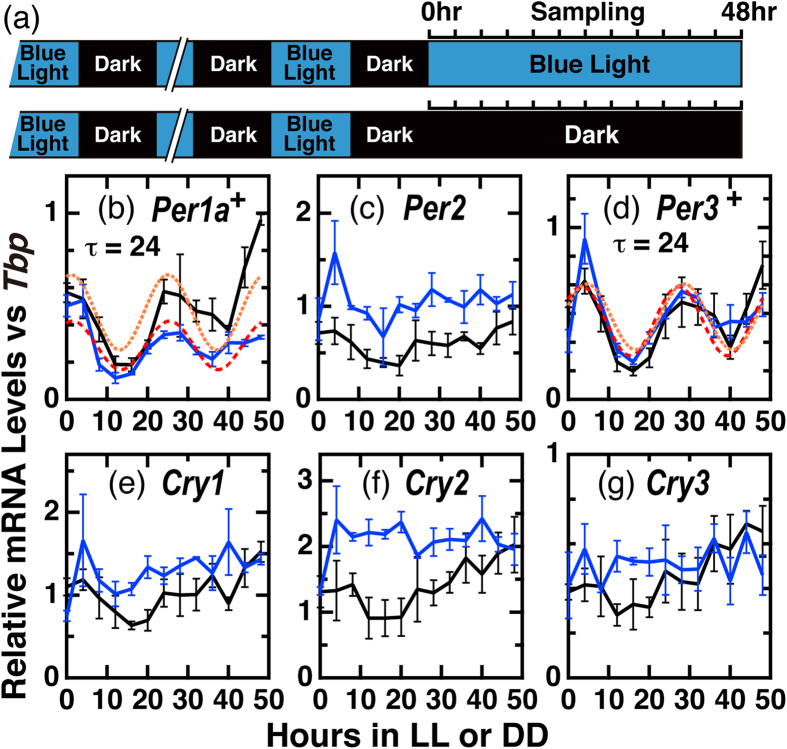
Temporal profiles of mRNA levels of *TrPer* and *TrCry* genes in LL by qRT-PCR analysis. (**a**) Schematic representation of the experimental design. Fugu eye cells in culture dishes (n = 3) were entrained to blue light (100 μW/cm^2^)/dark cycles for 4 days and maintained under LL/DD conditions for 48 h. (**b–g**) qRT-PCR of mRNA levels of *TrPer* and *TrCry* genes in Fugu eye cells under constant blue-light (LL; blue lines) or dark (DD; black lines) conditions. Intensity of blue light: 100 μW/cm^2^ (3.9 μmol photons/s/m^2^). In each panel, points are plotted as the means of three independent experiments +/−SE.^+^p < 0.01 indicate significant rhythmicity with a 24 h period in both LL and DD (JTK Cycle, Table S2). Red dashed and orange dotted lines in panels (**b**) and (**d**) denote CircWave cosine-fitting curves for DD and LL data, respectively.

Along with the *TrPer1a* and *TrPer3* identified in Group II (Fig. 1), we additionally analyzed the mRNA expression profiles of the four clock genes *TrPer2, TrCry1, TrCry2*, and *TrCry3* by qRT-PCR (Fig. 4). We tried to detect the mRNA expression of *TrHspa1l* in Group II, but its cDNA was not amplified from Fugu eye cDNA (Table 1). Messenger RNA levels of *TrPer2* and *TrPer3* reached the maximum after 4 h light exposure then rapidly decreased (Fig. 4), and *TrPer2* displayed weak (1.74 fold) but significant upregulation by light during the 8–48 h period (Table 3). Rhythmicity analyses (Table S2) and Cosinor-fitting analyses (Table 4) showed that *TrPer1a* and *TrPer3* mRNA levels exhibited clear circadian oscillations with a period of 24 h and peaks in the morning (CT1.1-CT4.6). *TrCry1* and *TrCry2* showed weak upregulation by light during the 8–48 h period (1.30 and 1.51 fold, respectively, Table 3). *TrCry3* mRNA levels showed no significant circadian oscillation or response to light (Fig. 4g, Tables 2, 3 and S2). These results indicate that the circadian clock continuously oscillated under both LL and DD conditions in the Fugu eye cells.

**Figure 5:**
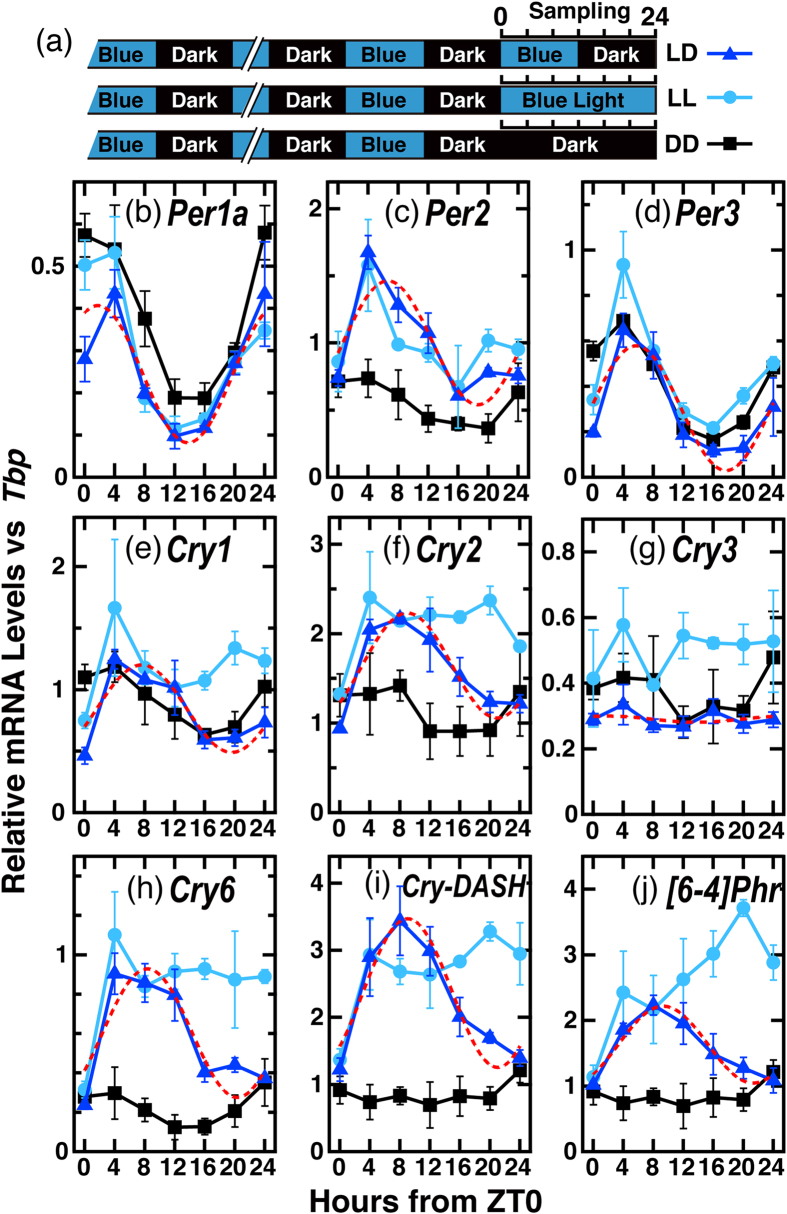
Messenger RNA levels of clock-related and photolyase genes in Fugu eye cells under various light conditions. (**a**) Schematic representation of the experimental design. Fugu eye cells in culture dishes (n = 3) were entrained to blue light (100 μW/cm^2^)/dark cycles for 4 days and maintained under LD/LL/DD conditions. (**b–j**) qRT-PCR analysis of mRNA levels of clock-related and photolyase genes in Fugu eye cells under blue light/dark (LD, deep blue triangles) or constant dark (DD, black squares) or constant blue light (LL, blue circles) conditions. Data for LL and DD are reproduced in part (0–24 h) from that presented in Fig. 3 (*TrCry-DASH, TrCry6*, and *Tr[6-4]Phr*) or Fig. 4 (*TrPer1a, TrPer2, TrPer3, TrCry1, TrCry2*, and *TrCry3*). Intensity of blue light: 100 μW/cm^2^ (3.9 μmol photons/s/m^2^). In each panel, points are plotted as the means of three independent experiments +/−SE. Red dashed curves denote cosine-fitting to data for LD (calculated by CircWave1.4; Table 4).

We next examined expression profiles of the clock(-related) genes under LD conditions. These data were merged in Fig. 5 with the profiles in LL (0–24 h) and DD (0–24 h) reproduced from Fig. 3 and Fig. 4 in order to evaluate the regulation by light and the circadian clock. *TrPer1a* and *TrPer3* showed oscillatory patterns regardless of the light condition (Fig. 5b and d), suggesting that light during the night period (ZT12-ZT24) may not affect their expression. *TrCry1, TrCry2, TrCry6, TrCry-DASH*, and *Tr[6-4]Phr* showed relatively higher mRNA expression levels in LD during the light period (ZT4-ZT12) but the expression levels decreased under dark conditions (ZT16-ZT24), showing a more dominant regulation of these genes by light rather than the circadian clock (Fig. 5). The daily variations of mRNA levels of all the examined genes except for *TrCry3* were statistically significant (p < 0.0001, Table 4).

### Wavelength dependency of light-induced gene expression

**Figure 6:**
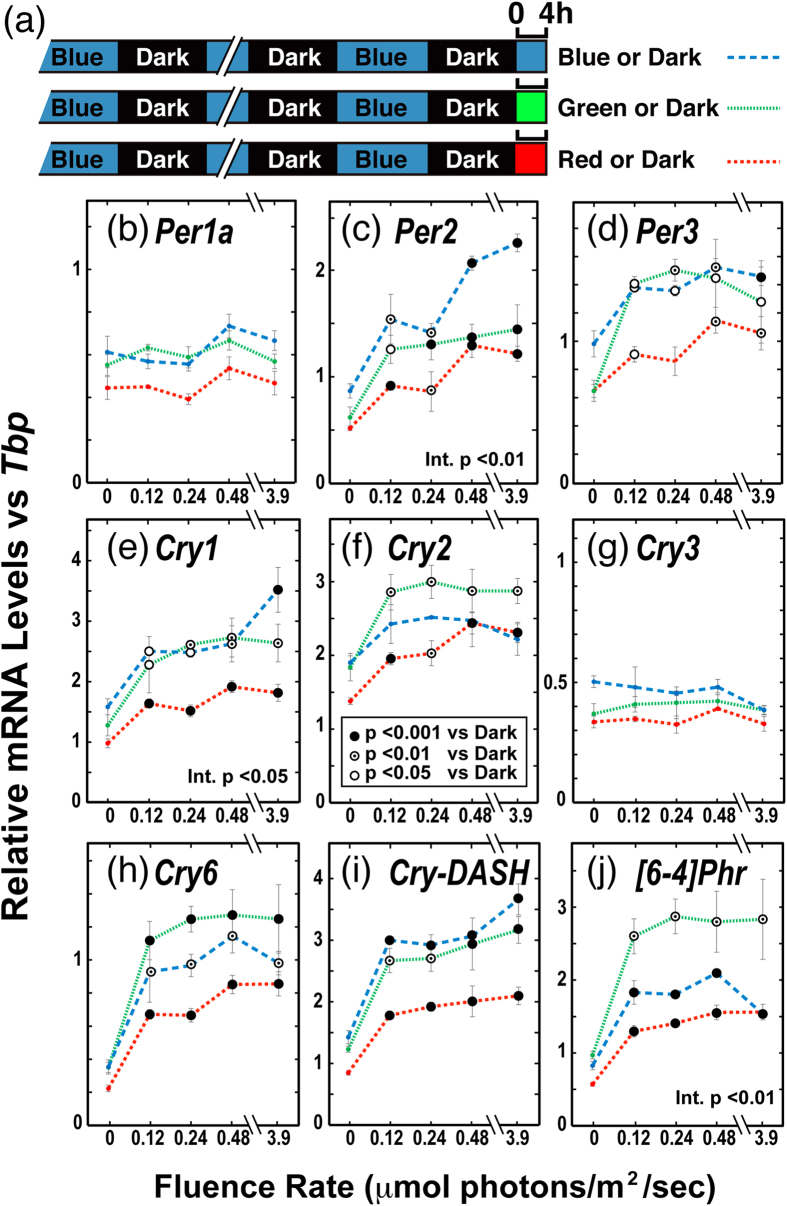
Spectral dependency and dose dependency in the light-dependent mRNA upregulation of clock-related and photolyase genes. Fugu eye cells in culture dishes (n = 3) were entrained to blue light (100 μW/cm^2^)/dark cycles for 4 days and irradiated with various intensities of blue (Philips Lumileds, LXK2-PR14-Q00; λmax = 462 nm; λ_1/2_ = 20 nm; blue dashed lines), green (Philips Lumileds, LXK2-PM14-U00; λmax = 539 nm; λ_1/2_ = 33 nm; green dotted lines), or red (Epistar, 33R-Y1-1; λmax = 654 nm; λ_1/2_ = 15 nm; red lines) light (0–3.9 μmol photons/s/m^2^) for 4 h from ZT0 (Fig. S2). The maximum intensity of blue LED light (3.9 μmol photons/s/m^2^) corresponds to 100 μW/cm^2^. Error bars represent standard deviation.

As the first step in elucidating the photopigment contributing to light-induced gene expression, we examined whether green or red light could trigger the acute light response of mRNA levels like blue light did. *TrPer, TrCry*, and *Tr[6-4]Phr* genes were examined after 4 h light exposure to different fluence rates of red, green, or blue light (Fig. 6). The mRNA levels of all examined genes except for *TrPer1a* and *TrCry3* were upregulated by not only blue light but also green and red light. These genes showed significant light-dependent increases in mRNA levels at 0.12 μmol photons/s/m^2^. Two-way ANOVA indicated significant interaction among the light sources in *TrPer2, TrCry1* and *Tr[6-4]Phr*. However, these interactions were likely due to experimental error since the light irradiation was performed in different incubators and there were differences in the expression levels in the dark (zero fluence rates for different color conditions).

### Light-induced gene expressions are not induced by heat-treatment

**Figure 7:**
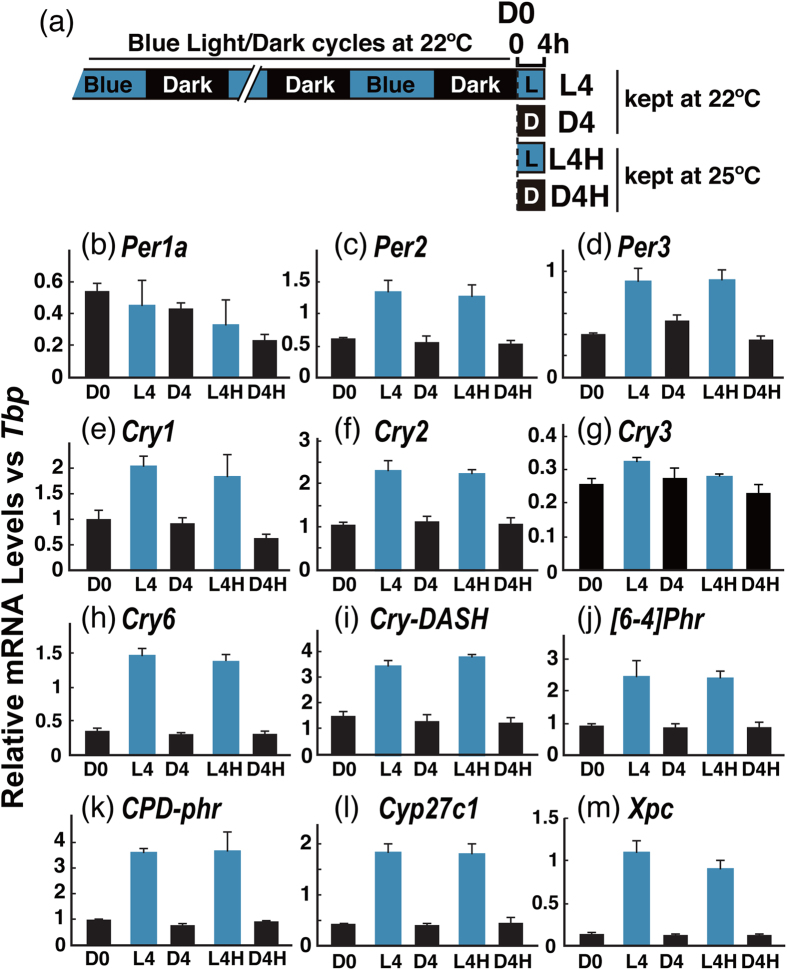
Heat-treated Fugu eye cells. Fugu eye cells were entrained to blue light/dark cycles for 4 days and then harvested before and after blue-light (100 μW/cm^2^) and/or heat (25 °C) treatment for 4 h. D0, before the treatment (ZT0); L4, after 4 h blue-light treatment at 22 °C; D4, kept in the dark for 4 h at 22 °C; L4H, after 4 h blue-light treatment at 25 °C; D4H, kept in the dark for 4 h at 25 °C. There was no significant difference between 22 °C and 25 °C (L4 vs L4H or D4 vs D4H). In all examined genes except for *Per1a* and *Cry3* (a light-insensitive gene), L4 and L4H were significantly higher than D4 and D4H, Error bars represent the standard deviation.

Although we ensured that the culture media were kept at a constant temperature (22 ± 0.3 °C) during the light irradiation in this study, we tested the effect of heat and whether increased cell temperature would affect gene expression. We transferred the culture flasks from 22 °C to 25 °C, and mRNA levels of the light-responsive genes (L4H, D4H, Fig. 7) were compared with those genes kept at 22 °C (L4, D4, Fig. 7). We examined the mRNA levels of 12 genes, including light-insensitive genes *TrPer1a* and *TrCry3*. The light-responsive genes examined were induced by light at both 22 °C (L4 vs D4) and 25 °C (L4H vs D4H), and no significant difference was observed under light (L4H vs L4) or dark (D4H vs D4) conditions at the different temperatures (Int. p > 0.089, Int. q > 0.45). [Fig f8]

## Discussion

In the present study, we identified genes that are controlled by the circadian clock and/or external light signals by analyzing the temporal changes in transcript levels during light-irradiation of Fugu eye cells using microarray (Fig. 1) and qRT-PCR analysis (Figs 3, 4, 5).

The Group I genes showed blue light-dependent upregulation of their transcripts with similar temporal profiles, except for *Tr[6-4]Phr* (Fig. 3 and Table 3). Messenger RNA levels of *Tr[6-4]Phr* seemed to continuously increase under 48 h light irradiation (Fig. 3i). Interestingly, the mRNA levels of *TrCry* genes did not oscillate under either LL or DD conditions, yet most appeared to respond to light (Figs 4, 5, 6). This is different from *Cry* mRNA levels that oscillate in a circadian manner in mammals^31^ and play a pivotal role in clock oscillation^32^. In contrast to *Cry* mRNA, *TrPer1a* and *TrPer3* mRNA levels displayed circadian oscillation under both LL and DD conditions (Fig. 4b and d). Such differences likely stem from the combination of cis-elements in their promoters; CRE, RORE, D-box, E-box and G-box elements may contribute to the gene expression induced by light and/or circadian information^6^,^16^,^19^,^22^,^23^,^24^,^25^,^33^.

**Figure 8 f8:**
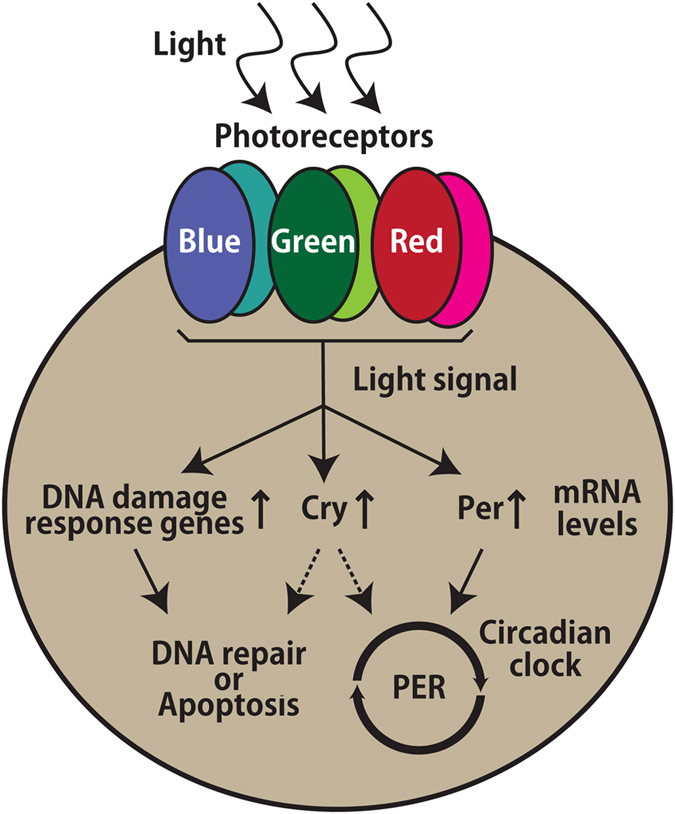
Model for photopigments and intracellular regulatory pathways in Fugu eye cells.

Because the light-induced gene expression is not induced by heat-treatment (Fig. 7), thermal induction could be ruled out under the present conditions. Photic induction was exerted by not only blue but also green and red light (Fig. 6). Based on the spectrum of photic responses observed, we were able to speculate on the underlying molecular mechanism (Fig. 8). Blue light has been shown to generate reactive oxygen species (ROS) as well as ultraviolet radiation, while yellow and red light have not^13^. In the case of CPD-Phr activity in goldfish RBCF1 cells and *Per2* mRNA expression in Z3 cells, green and blue light but not yellow light have been shown to have the inducibility^15^,^34^, while the presence of an ROS-dependent pathway has been suggested in Z3 cells^15^,^20^. On the contrary, Fugu eye cells (Fig. 6) and PAC-2 cells^24^ were sensitive to red light, and therefore an ROS-independent mechanism is likely in operation.

Blue light is known to trigger photooxidative damage in retinal cells through the photoactivation of blue-light-absorbing compounds such as lipofuscin A2E^35^. It is important to note that both red light and blue light induced *TrCry-DASH* and *Tr[6-4]Phr* genes in Fugu eye cells (Fig. 6i and j). A protective response to intense blue light or UV radiation may be induced by the longer wavelength light in the Fugu eye cell. The origin of Fugu eye cells has yet to be precisely identified. The wide spectral sensitivity suggests that Fugu eye cells may have originated from an unidentified ocular cell expressing multiple photopigments or one that became differentiated during establishment of the cell line.

Opsin is the most plausible candidate involved in photic induction in Fugu eye cells due to its broad spectral sensitivity that spans the blue to red regions. The contribution of opsin is also suggested based on the high sensitivity observed in the Fugu eye cell. In this study, the light-induced genes in Fugu eye cells were significantly upregulated only at a dose of 0.12 μmol photons/s/m^2^ (Fig. 6), which corresponds to approximately 3 μW/cm^2^ (1/45,500 of the solar constant). Therefore, it is possible that the photic signals from multiple opsins, such as blue, green, and red visual opsins, are integrated to contribute to the photic gene expressions (Fig. 8), though a novel photopigment with a broad spectral sensitivity cannot be ruled out. Besides visual opsins, non-visual opsins such as TMT opsin^36^ and melanopsins (Opn4s)^37^ are strong candidates contributing to photoreception, because of their likely involvement in the circadian physiology of the blind cavefish-derived CF cell^38^. Recent reports consistently suggest that, in zebrafish, 42 opsin genes exist in the genome and more than 10 opsin genes are transcribed in the eye^39^. Thus, it is important to investigate the endogenous expression of visual opsins, non-visual opsins, and cryptochromes in the Fugu eye cell.

Several photoreceptive cell lines have been reported to date, but they have been derived from only a few fish species^7^,^13^,^14^,^18^,^21^,^38^. Here we add a puffer fish cell line to the cell models for photobiology and circadian biology. To our knowledge, other cell models in the circadian biology field have originated from animals of lesser commercial value. The Fugu eye cell is the first model cell line from a marine fish that also has special importance as a food resource. Analyses of clock gene expressions have been reported in edible marine fish species, i.e. the Senegalese sole^40^,^41^ and gilthead sea bream^42^. Considering the close evolutionary kinships between Fugu and these marine fishes, analyses of the circadian clock machinery of Fugu eye cells might help to understand the circadian physiologies of marine fishes.

The photic response of clock genes and their transcriptional regulation have been extensively studied in zebrafish photosensitive cells^14^,^15^,^16^,^18^,^19^,^20^,^21^,^22^,^23^,^24^,^25^,^26^. In addition, *Xpc, Lonrf1*, and *Neil1*, which were identified as light-responsive genes in the Fugu eye cells (Tables 2 and 3), have been reported as light-responsive genes in zebrafish^23^,^43^, suggesting conserved mechanisms across species. In zebrafish, the ERK signaling cascade likely operates in the photic induction of *zCry1a* through a D-box in its promoter, which likely plays a key role in the circadian clock-phase resetting by acting on the *zPer1* promoter^19^,^26^,^43^. Most techniques described in studies using zebrafish photosensitive cells can be utilized to analyze the photic regulatory mechanisms underlying light-responsive clock genes in Fugu eye cells. Comparative studies of the Fugu eye cell as well as the other photosensitive cells would help to clarify the species-specificity and generality of the photic gene regulation mechanisms.

As is well known, the genome size of the puffer fish is relatively small, making the Fugu eye cell suitable for genome-wide analysis of the gene regulatory circuits sustaining circadian oscillatory transcription. The compactness of the genome is useful in genome editing, such as for functional disruption using CRISPR/Cas9, because the possibility of being off target geometrically decreases with reduced genome size. In addition, the copy numbers of *Cry* orthologs are fewer in puffer fish than in other fish; i.e. the zebrafish has four orthologs of animal-type CRY1 genes (*zCry1a/1b/2a/2b*), while the puffer fish has one (*TrCry1*).

In many marine fishes, the lunar and tidal cycles have significant impact on gonadal development and spawning behavior^44^. The present identification of clock genes and their expression in Fugu eye cells may have relative scientific importance from the viewpoint of chronobiology: Grass puffer, *Takifugu niphobles,* is very closely related to *Takifugu rubripes* and shows a robust semi-lunar spawning rhythm during reproductive season (from spring to summer in Japan)^45^. Taking into consideration the presence of a circadian clock in other cultured cell lines, Fugu eye cells may contain a lunar or semi-lunar clock oscillator, although molecular and/or cellular mechanisms underlying the presence of a lunar or semi-lunar clock remain unclear. In this regard, we have previously speculated on the presence of clock genes and components of a lunar clock oscillator based on the observation that *Cry* and *Per* mRNA levels oscillate in a lunar-phase dependent manner in the hypothalamus of goldlined spinefoot, a tropical fish showing lunar-dependent spawning rhythms^46^,^47^. Future analyses of circadian- and light-controlled gene expression in Fugu eye cells under various light conditions, such as different light intensities, spectra, and photoperiodic regimes, may help to better understand not only the circadian physiology of marine fishes but also the presence of a lunar or semi-lunar clock oscillatory mechanism(s).

## Materials and Methods

Experiments were conducted in accordance with the guidelines of WASEDA University, and the experimental protocols were approved by the Committee for the Management of Biological Experiment at WASEDA University (permission # WD14-002, WD15-060). The Fugu eye cell line (ATCC:CRL-2641) was maintained according to a method described previously^28^. The cells were maintained in 60% Dulbecco’s modified Eagle medium (DMEM, Sigma, powder) and 25% L-15 medium (Gibco) and 15% DMEM/F-12 medium (Gibco) supplemented with 15 mM HEPES, 0.5 g/L sodium bicarbonate, 2.7 g/L D-glucose, 0.01 mg/mL bovine insulin (Gibco), 0.05 mM beta-mercaptoethanol (Nacalai Tesque), 5 mM L-glutamine (SIGMA), 0.05 mM non-essential amino acids (Invitrogen-Gibco), 10 ng/mL basic fibroblast growth factor (bFGF, Sigma-Aldrich), 50 ng/mL epidermal growth factor (EGF, Sigma-Aldrich), and 7.5% (v/v) fetal bovine serum (FBS, Hyclone).

Cultures were replated at a 1:2 dilution once reaching confluence. The culture medium was aspirated and the cell layers were rinsed with PBS (10 mM sodium phosphate (pH 7.4), 140 mM NaCl, 1 mM MgCl_2_) containing 0.1% EDTA several times. The cells were dissociated in PBS containing 0.25% trypsin and incubated at room temperature until the cells detached. To inactivate trypsin, complete culture medium was added to a sealed 25-cm^2^ flask (IWAKI) and resuspended by gently pipetting. Cell suspension was transferred to a centrifuge tube and centrifuged (160 x g, 10 min). Supernatants were aspirated and the pellet was resuspended in fresh culture medium by gently pipetting. The resuspended cells were cultured at 22 °C without CO_2_.

For light-entrainment, the Fugu eye cells were plated at a density of 1.0~2.0 × 10^6^ cells/25 cm^2^ flask with phenol-red-free DMEM/L15/FBS with all the supplements. The Fugu eye cells were transferred to a light-irradiation apparatus in the incubator (see below) and entrained to a 12 h light/12 h dark cycle (LD) for 4 days. Unexposed control cells were also processed in a similar manner, with only the light treatment omitted.

### LED and Light Irradiation

Each light-irradiation apparatus was constructed using light–emitting diodes (LED, Philips Lumileds LUXEON K2 or Epistar, Fig. S2). Unless otherwise specified, the intensity of blue-light LED (λ_max_ = 462 nm) corresponded to 100 μW/cm^2^ (3.9 μmol photons/s/m^2^). The LEDs were driven by direct current using a power supply (LX035, Takasago, Japan). In the incubator, cells were irradiated through the top of the culture flask. The temperature of the culture medium was maintained at 22 ± 0.3 °C during the 4-h irradiations except for the heat treatment experiment.

### Microarray Analysis

The LD entrained Fugu eye cells were harvested at ZT0 (D, Dark, before blue light stimulation) or after blue light (3.9 μmol photons/s/m^2^, 100 μW/cm^2^) stimulation for 4–20 h (L4, L8, L12, L16, L20). Total RNA was extracted using TRIzol reagent (Invitrogen) according to the manufacturer’s instructions. RNA was purified using the DNase (RNase-free DNase I Set, QIAGEN) and RNeasy mini kit (QIAGEN). cRNA labeling, hybridization, and washing was performed using the Low Input Quick Amp Labeling Kit (Agilent Technologies). For each time point, two RNA pools from two independent culture plates were analyzed by using two independent arrays on different slides in a 4 × 44 k format. A custom microarray of 60 base oligonucleotides was designed by the Agilent eArray program to contain 19,877 predicted transcripts (2 or 3 different probes per transcript in duplicate spots) based on the Fugu genome database (Fugu ver.5 release ensemble as of Dec. 2013). Data scanning was performed by Medical and Biological Laboratories Co. Ltd. (Japan).

Background correction, normalization and averaging of the signal intensities, and further analyses were performed by using R (R ver. 3.02)^48^ and Bioconductor^49^; Limma package v.3.18.13)^50^. Raw data for each transcript at the same time point were processed to obtain normalized expression levels with the ‘normalizeBetweenArrays’ option. Genes showing possible significant (p < 0.01) changes were deduced using the Benjamini-Hochberg method. Expression profiles for the selected genes were further analyzed by clustering them according to cosine distance (cosine similarities) for each pair of the six-dimensional vectors for two data sets (values for ZT0 and L4-L20 as components) by using the Proxy (ver. 0.4.12)^51^ and Gplots (ver. 2.13)^52^ packages.

### Quantitative RT-PCR Analysis

The cultured cells were washed with PBS (10 mM sodium phosphate (pH 7.4), 140 mM NaCl, 1 mM MgCl_2_), homogenized in 2 mL TRIzol reagent (Invitrogen), and stored at −80 °C. The extraction of total RNA and quantitative real-time PCR (qRT-PCR) analysis were carried out as described previously^53^. In brief, total RNA was extracted using TRIzol reagent (Invitrogen) according to the manufacturer’s instructions. Residual genomic DNA in the total RNA sample was eliminated by DNase I treatment (RNase-free recombinant DNase I, TaKaRa). Complementary DNA was prepared using a high capacity cDNA reverse transcription kit (Applied Biosystems). Each reaction included 1 μg of total RNA.

The pairs of primers shown in Table 1 were designed for cDNA cloning and qRT-PCR analyses of genes selected by the microarray analysis. Sequences of the qRT-PCR primers were confirmed by sequencing of the cloned cDNAs by using the cDNA cloning primers. Additionally we prepared primer pairs for *TrPer2* (CACTGGCGCAGAGTCCTCTC and CGCTCCTTGCCACCACTG), *TrCry1* (AGCAGCTCTCCTGCTACAGAGG and GTGAACGCTCTGCTTACCGG), *TrCry2* (GTCAACAGGTGGCGGTTTCT and CCGTGAGATCTTCCATTCCTTAAA), and *TrCry3* (CTGCGCTGCATCTACATCCT and TCACCTTCCAGTCCTTCAGC) based on the Fugu genome database. The StepOnePlus System (Applied Biosystems) was employed using the FAST SYBR Green PCR master kit (Applied Biosystems) according to the manufacturer’s recommendations with the following cycle conditions: 20 s at 95 °C, then 40 cycles of 3 s at 95 °C, and 30 s 60 °C, and then 15 s at 95 °C, 60 s 60 °C and 15 s 95 °C (melting curve analysis). The PCR products were subjected to 3% agarose gel electrophoresis and analyzed using a Typhoon 9410 scanner (GE healthcare). The relative levels of each mRNA were calculated by the 2^−ΔΔCT^ method. We first elected to use *TrTbp* (TATA-binding protein; ENSTRUG00000007200), *TrHprt1*, and *TrB2m* (beta-2 microglobulin) as candidates for the reference gene, and *TrTbp* (primers; GCAGAATACAATCCAAAGCGTTT and CTAACCTTGACTGCTCCTCACTCTT) was selected for normalization of the expression levels in all the experiments because of its mostly constant mRNA levels under both LD and DD conditions. Each CT value is the mean of three biological replicates.

### Statistics

The significance of light responsiveness was analyzed by one-way or two-way ANOVA and Turkey-Kramer post hoc test. The significance of circadian rhythmicity was analyzed by JTK Cycle^54^ and CircWave (Ver.1.4) by R. Hut (available at http://www.euclock.org/results/item/circ-wave.html). Multiple comparison-testing corrections were done using the Benjamini-Hochberg (BH) method^55^. Where indicated, two experimental data sets were compared using a two-tailed Student’s *t* test.

## Additional Information

**How to cite this article**: Okano, K. *et al*. Light- and circadian-controlled genes respond to a broad light spectrum in Puffer Fish-derived Fugu eye cells. *Sci. Rep.* **7**, 46150; doi: 10.1038/srep46150 (2017).

**Publisher's note:** Springer Nature remains neutral with regard to jurisdictional claims in published maps and institutional affiliations.

## Supplementary Material

Supplementary Information

## Figures and Tables

**Table 1:** Blue light-responsive genes identified in microarray analysis of Fugu eye cells and primers used in the cDNA cloning and quantitative RT-PCR analysis.

Group	Ensembl Transcript ID	Gene Name	Description	Cloning primers (Forward/Reverse)	qRT-PCR primers (Forward/Reverse)
Group I	ENSTRUT00000013152	*Lonrf1*	LON peptidase N-terminal domain and ring finger 1	CCTGCAAAAGGCCAAATCA	CCCTGTGCTAAAAGACACGTAGAAA
				CAGAGAGGACAGCGGAGGTT	AAGTGAGGCGATGAGTTGTGG
	ENSTRUT00000027195	*Xpc*	repair damaged DNA	CTGATGGCTACGTTGTTTGTGA	CCTGGGTGGAAGAGCAAGA
				GTCCTCCTCTTCTTGTCTGTTCTG	CCCTTTACCAGCAGAGTCCAA
	SINFRUT00000129951	*Cry6*	termed as Cry6 in this study	CCTTACTCTGTCAGTACAGAGG	GGAAGGACGGCACAACGAT
				CACAATAACCAGCGTGGGCT	CTGTACACACCGTCATGAAGGAA
	ENSTRUT00000000813	*Hells*	Helicase	GTGGTGAGCTATAAAGAAACGGAT	GAGGACTTGGAATCAACCTGAC
				CTTCGACACTTTCATTTCACACTC	TCCCACCTTTGAACTTGTTCTT
	ENSTRUT00000044170	*Cyp27c1*	Cytochrome P450	CAAACCGTGGGAGGAGTTCT	GAGATGAGCGTCGAGGAGATTTA
				CATCCTGCGTAATTCGACCA	CTCTGCGTGGATTTGTTGTTGTA
	ENSTRUT00000034552	*Neil1*	nei-like DNA glycosylase 1	CAGAGTGCTGAGTTTTGTGGAC	CAACTACCTGAGGGCTGAAATC
				CCTCTTTTTCACCTTTGGTGTC	ACCACCTCCAGAGGTATTGTGT
	ENSTRUT00000022159	*CPD-Phr*	deoxyribodipyrimidine photolyase	CTTGGCTCCACGTTTCGTC	CCACTTTGAAAACGCTCAAACA
				CATCTCCTTGTTTTCCGTCCTC	GTGAGTAACAGCCAGCCACAAA
	ENSTRUT00000008943	*[6-4]Phr*	photoreactivating enzyme	GTGACAAGGCTGGCCGAA	CTCTATGGAGGATTTGAAAGATGTGA
				CAGACAGTCTCCACCAGAAG	GCAAACCCAAGCCGTTCTT
	ENSTRUT00000038403	*Dhrs12*	SDR family member 12	CTGCAGGAGTACACCAAGAGTG	TGCTACAAATACACTTGGCACC
				CTGCTGTCTTTTGTTCTGAGCAT	CACGTTGAGCTTTTGTGTAAGC
	ENSTRUT00000044961	*Cry-DASH*	Cryptochrome DASH	CCCAGATGAAGGTCAACGTTC	GGACCTGGAACAGAAAGAGCC
				GGAGAAATGCAACCCAACCG	CTTTGTAAACTGCGACTGCGTCT
	ENSTRUT00000017713	*Sdhb*	succinate dehydrogenase complex, subunit B	CATTGAGCCCTACCTGAAGAAGA	GCTACTGGTGGAATGGGGATAA
				GCCTTTCCTGGGTTGAGTCC	CCAGACGCTCCTCGGTAAA
	NM_001146062.1	*Igfbp4*	insulin-like growth factor binding protein 4	not amplified	not tested
Group II	ENSTRUT00000007589	*Per3*	Period 3	GTGTGTGTAGCAGCGAGAAC	GCGTTAAACAAGTCCAAGCTAACA
				GCCGTGTGTGAGTAGAACAC	CACCACAAAGGAGTCCGTGTT
	ENSTRUT00000037001	*Per3*	Period 1a	CTGCAGCAGCAACCAGTCA	CACTCCGGATACGAAGCTCCT
				CCGCTGTAGAAGGTGCTGA	GGCACCGCCCTTTCGT
	ENSTRUT00000052058	*Hspa1l*	heat shock 70 kDa protein 1-like	not amplified	not tested
	ENSTRUG00000000024			not amplified	not tested

**Table 2:** Student’s *t* test of mRNA expression levels of 17 genes after incubation of Fugu eye cells for 4 h or 8 h under blue light (L4 and L8) or dark (D4 and D8) conditions.

Gene	t-test L4,D4		t-test L8,D8		Figure
	p-value^+^	q-value(BH)	p-value^+^	q-value(BH)	
*Lonrf1*	0.0624	0.0624	**0.0215**	0.0430*	3b
*Xpc*	0.0582	0.0582	**0.0047**	0.0094**	3c
*Cry6*	**0.0395**	0.0395*	**0.0062**	0.0124*	3d
*Hells*	**0.0280**	0.0280*	**0.0084**	0.0167*	3e
*Cyp27c1*	**0.0130**	0.0130*	**0.0022**	0.0044**	3f
*Neil1*	**0.0245**	0.0245*	**0.0107**	0.0214*	3g
*CPD-Phr*	**0.0081**	0.0081**	**0.0053**	0.0081**	3h
*[6-4]Phr*	0.0715	0.0715	**0.0262**	0.0525	3i
*Dhrs12*	**0.0396**	0.0396*	**0.0030**	0.0060**	3j
*Cry-DASH*	**0.0302**	0.0302*	**0.0143**	0.0286*	3k
*Sdhb*	**0.0043**	0.0049**	**0.0049**	0.0049**	3l
*Per1a*	0.9793	0.9793	**0.0316**	0.0631	4b
*Per2*	0.0894	0.0894	0.0599	0.0894	4c
*Per3*	0.1801	0.3602	0.4551	0.4551	4d
*Cry1*	0.2979	0.2979	0.1944	0.2979	4e
*Cry2*	0.1344	0.1344	**0.0301**	0.0603	4f
*Cry3*	0.1088	0.2177	0.8723	0.8723	4g

^**+**^p-values indicate the probability of no difference between light and dark.

^*,**^q-values are p-values adjusted for multiple comparisons of data from two time points using the BH method.

^*^q < 0.05; ^**^q < 0.01.

**Table 3:** ANOVA of mRNA expression levels of 17 genes after incubation of Fugu eye cells for 8–48 h under blue light or dark conditions.

Gene	Two-way ANOVA				Expression levels vs *Tbp*				LL/DD Ratio	Figure				
	LL (8–48 h), DD (8–48 h)				LL (8–48 h)		DD (8–48 h)							
	Interaction p	q-value(BH)	LL vs DD (p)	q-value(BH)	Average	SE	Average	SE						
*Lonrf1*	0.6923	0.7356	**5.03.E-26**	9.50.E-26**	12.23	0.36	3.18	0.14	3.85	3b				
*Xpc*	0.2500	0.3542	**4.12.E-29**	1.40.E-28**	0.97	0.03	0.13	0.01	7.72	3c				
*Cry6*	0.0545	0.1029	**2.81.E-30**	1.59.E-29**	0.97	0.03	0.23	0.01	4.17	3d				
*Hells*	0.2797	0.3658	**2.63.E-26**	5.58.E-26**	0.61	0.02	0.14	0.01	4.31	3e				
*Cyp27c1*	0.5995	0.6794	**2.18.E-25**	3.70.E-25**	1.48	0.04	0.50	0.02	2.99	3 f				
*Neil1*	**0.0285**	0.0606	**9.26.E-27**	2.25.E-26**	3.76	0.17	0.41	0.02	9.15	3 g				
*CPD-Phr*	0.8789	0.8789	**2.70.E-31**	2.30.E-30**	3.88	0.10	0.62	0.03	6.27	3 h				
*[6-4]Phr*	**3.54.E-04**	0.0020**	**1.13.E-28**	3.20.E-28**	3.09	0.11	0.96	0.04	3.21	3i				
*Dhrs12*	0.5986	0.6794	**6.21.E-24**	9.59.E-24**	7.62	0.30	1.41	0.08	5.40	3j				
*Cry-DASH*	**0.0019**	0.0066**	**4.32.E-34**	7.35.E-33**	3.06	0.07	0.68	0.04	4.48	3k				
*Sdhb*	0.2241	0.3464	**8.08.E-30**	3.43.E-29**	11.70	0.38	1.37	0.08	8.55	3 l				
*Per1a*	**4.26.E-09**	7.24.E-08**	**1.43.E-16**	2.03.E-16**	0.26	0.01	0.47	0.04	0.55	4b				
*Per2*	0.1259	0.2140	**2.07.E-16**	2.71.E-16**	1.02	0.03	0.59	0.03	1.74	4c				
*Per3*	**0.0099**	0.0239*	0.3045	0.3045	0.46	0.02	0.44	0.03	1.05	4d				
*Cry1*	**0.0011**	0.0048**	**7.40.E-09**	8.38.E-09**	1.31	0.04	1.01	0.05	1.30	4e				
*Cry2*	**2.67.E-05**	2.27.E-04**	**8.73.E-15**	1.06.E-14**	2.14	0.04	1.42	0.08	1.51	4 f				
*Cry3*	**0.0055**	0.0156*	0.0951	0.1010	0.51	0.02	0.47	0.03	1.09	4 g				

^*,**^q-values are p-values adjusted for multiple comparisons of the 17 genes using the BH method.

^*^q < 0.05; **q < 0.01.

**Table 4:** Cosinor analysis of rhythmicity of mRNA expression levels of *Per, Cry*, and *Cry*-related genes by CircWave1.4.

Gene	Light	Acrophase	p-value	Mean	SD	COG*	SD	Figure		
*Per1a*	LD	1.8	**6.10E-06**	0.27	0.14	0.98	2.06	5b		
	LL	1.4	**2.00E-07**	0.30	0.13	0.92	2.67	4b		
	DD	1.1	**1.86E-05**	0.48	0.22	0.69	2.69	4b		
*Per2*	LD	6.7	**1.96E-05**	0.99	0.37	4.19	2.98	5c		
*Per3*	LD	5.7	**1.10E-06**	0.31	0.21	4.30	2.21	5d		
	LL	3.3	**2.50E-06**	0.49	0.19	3.18	2.95	4d		
	DD	4.6	**1.30E-06**	0.47	0.20	2.24	2.78	4d		
*Cry1*	LD	7.6	**3.12E-05**	0.83	0.30	4.98	3.15	5e		
*Cry2*	LD	9.0	**1.20E-06**	1.59	0.48	6.00	3.39	5 f		
*Cry3*	LD	n.d.	0.721	0.29	0.04	0.15	3.21	5 g		
*Cry6*	LD	8.4	**5.90E-06**	0.58	0.27	6.71	3.05	5 h		
*Cry-Dash*	LD	9.0	**4.00E-07**	2.25	0.88	7.26	3.22	5i		
*[6-4]Phr*	LD	9.3	**6.00E-07**	1.57	0.48	6.53	3.42	5j		

^*^COG, center of gravity.
