# Supplementary material for: Light- and circadian-controlled genes respond to a broad light spectrum in Puffer Fish-derived Fugu eye cells
Source: Sci Rep. 2017 Apr 18;7:46150. doi: 10.1038/srep46150 (PMC5394683; doi:10.1038/srep46150)
Supplement: Supplementary Information [file srep46150-s1.pdf]

Supplementary Information

Title:

Light- and circadian-controlled genes respond to a broad light spectrum in Puffer Fish-derived Fugu eye cells

Author List:
Keiko Okano[1], Shoichi Ozawa[1], Hayao Sato[1], Sawa Kodachi[1], Masaharu Ito[1], Toshiaki Miyadai[2], Akihiro Takemura[3], and Toshiyuki Okano[1*]

Author affiliations:
[1] Department of Electrical Engineering and Bioscience, Graduate School of Advanced Science and Engineering, Waseda University, Wakamatsu-cho 2-2, Shinjuku-ku, Tokyo 162-8480, Japan

[2] Department of Marine Bioscience, Faculty of Marine Bioscience, Fukui Prefectural University, 1-1 Gakuen-cho Obama, Fukui 917-0003, Japan

[3] Department of Chemistry, Biology, and Marine Science, Faculty of Science, University of the Ryukyus, 1 Senbaru, Nishihara-cho, Nakagami-gun, Okinawa 903-0213, Japan

[*]**Corresponding author:**
Dr. Toshiyuki Okano
Department of Electrical Engineering and Bioscience, Graduate School of Advanced Science and Engineering, Waseda University, Wakamatsu-cho 2-2, Shinjuku-ku, Tokyo 162-8480, Japan
Tel and Fax: +81-3-5369-7316
E-mail: okano@waseda.jp

Table S1 ANOVA of mRNA expression levels of 17 genes after incubation of Fugu eye cells under light or dark condition for 0-48 h

Gene	Two-way ANOVA LL (0-48h), DD (0-48h)		One-way ANOVA LL (0-48h)		One-way ANOVA DD (0-48h)		Figure
	Interaction p	q-value(BH)	p-value	q-value(BH)	p-value	q-value(BH)	
Lonrf1	**1.92.E-06**	**4.29.E-06** **	**9.05.E-05**	**1.71.E-04** **	0.3475	0.3938	3b
Xpc	**1.64.E-05**	**2.64.E-05** **	**2.37.E-04**	**3.10.E-04** **	0.1525	0.2160	3c
Cry6	**6.60.E-07**	**1.87.E-06** **	**1.08.E-05**	**2.68.E-05** **	**0.0347**	0.0656	3d
Hells	**9.84.E-05**	**1.37.E-04** **	**2.21.E-04**	**3.10.E-04** **	**0.0203**	**0.0494** *	3e
Cyp27c1	**2.02.E-06**	**4.29.E-06** **	**1.27.E-05**	**2.69.E-05** **	0.4885	0.4885	3f
Neil1	**1.10.E-05**	**2.07.E-05** **	**2.30.E-04**	**3.10.E-04** **	0.3138	0.3938	3g
CPD-Phr	**8.49.E-08**	**3.61.E-07** **	**9.67.E-06**	**2.68.E-05** **	0.3941	0.4187	3h
[6-4]Phr	**1.66.E-07**	**5.64.E-07** **	**1.17.E-06**	**6.64.E-06** **	0.1039	0.1606	3i
Dhrs12	**0.0024**	**0.0027** **	**0.0094**	**0.0100** **	**0.0282**	0.0599	3j
Cry-DASH	**2.28.E-08**	**1.94.E-07** **	**1.11.E-05**	**2.68.E-05** **	0.0511	0.0869	3k
Sdhb	**5.37.E-08**	**3.04.E-07** **	**8.78.E-06**	**2.68.E-05** **	0.3375	0.3938	3l
Per1a	**7.87.E-10**	**1.34.E-08** **	**6.43.E-12**	**1.09.E-10** **	**1.38.E-09**	**2.34.E-08** **	4b
Per2	**0.0128**	**0.0128** *	**9.32.E-04**	**0.0011** **	**0.0033**	**0.0092** **	4c
Per3	**1.05.E-04**	**1.37.E-04** **	**1.54.E-11**	**1.31.E-10** **	**2.24.E-07**	**1.90.E-06** **	4d
Cry1	**2.49.E-04**	**3.02.E-04** **	**7.36.E-04**	**8.94.E-04** **	**2.90.E-05**	**1.64.E-04** **	4e
Cry2	**1.71.E-05**	**2.64.E-05** **	**1.63.E-04**	**2.78.E-04** **	**0.0021**	**0.0072** **	4f
Cry3	**0.0072**	**0.0077** **	0.0597	0.0597	**5.68.E-04**	**0.0024** **	4g

*,**q-values are p-values adjusted for multiple comparisons of the 17 genes using the BH method; *q < 0.05; **q < 0.01

Table S2 Analysis of rhythmicity of mRNA expression levels from 17 genes after incubation of Fugu eye cells under light or dark conditions for 0-48 h using the JTK-cycle.

Gene	JTK-cycle* LL(0-48h)					JTK-cycle* DD(0-48h)					Figure
	Period**	Acrophase**	p-value	q-value(BH)	Amplitude	Period**	Acrophase**	p-value	q-value(BH)	Amplitude	
Lonrf1	n.d.	n.d.	0.283	0.480	0.4820	n.d.	n.d.	1	1	n.d.	3b
Xpc	n.d.	n.d.	1	1	0.3538	n.d.	n.d.	1	1	n.d.	3c
Cry6	n.d.	n.d.	1	1	0.1040	n.d.	n.d.	0.224	0.762	n.d.	3d
Hells	n.d.	n.d.	0.064	0.219	0.0459	n.d.	n.d.	1	1	n.d.	3e
Cyp27c1	n.d.	n.d.	1	1	0.4017	n.d.	n.d.	1	1	n.d.	3f
Neil1	n.d.	n.d.	0.064	0.219	0.0460	n.d.	n.d.	1	1	n.d.	3g
CPD-Phr	n.d.	n.d.	0.192	0.362	0.9882	n.d.	n.d.	1	1	n.d.	3h
[6-4]Phr	**32**	**18**	**6.75.E-04**	**0.004**	0.6103	n.d.	n.d.	1	1	n.d.	3i
Dhrs12	n.d.	n.d.	0.150	0.362	1.0784	n.d.	n.d.	1	1	n.d.	3j
Cry-dash	n.d.	n.d.	1	1	0.3657	n.d.	n.d.	1	1	n.d.	3k
Sdhb	n.d.	n.d.	0.177	0.362	4.2794	n.d.	n.d.	1	1	n.d.	3l
Per1a	**24**	**2**	**1.35E-06**	**1.36E-05**	**0.1216**	**24**	**2**	**3.90E-05**	**3.32.E-04**	**0.1412**	4b
Per2	n.d.	n.d.	0.585	0.903	0.0468	n.d.	n.d.	0.117	0.589	n.d.	4c
Per3	**24**	**6**	**1.60E-06**	**1.36E-05**	**0.1467**	**24**	**4**	**4.41E-06**	**7.50E-05**	**0.1791**	4d
Cry1	n.d.	n.d.	1	1	0.1245	n.d.	n.d.	0.139	0.589	n.d.	4e
Cry2	n.d.	n.d.	0.150	0.362	0.2200	n.d.	n.d.	0.768	1	n.d.	4f
Cry3	n.d.	n.d.	1	1	0.0549	n.d.	n.d.	1	1	n.d.	4g

* Calculated between 16-36 h.

** Period lengths and acrophases were not determined (n.d.) when the q-value (p-value adjusted using the BH method) was greater than 0.05.

Figure S1
The alignment of CRY family proteins constructed by ClustalW2.1 (http://clustalw.ddbj.nig.ac.jp/) with default parameters.

```
hCRY1_NP_004066.1                   --------------------------------------------------
cCRY1_NP_989576.1                   --------------------------------------------------
XtCRY1_NP_001017311.2               --------------------------------------------------
TrCRY1a_XP_011611545.1              --------------------------------------------------
zCRY1a_CAH69047.1                   --------------------------------------------------
zCRY1b_NP_571865.4                  --------------------------------------------------
TrCRY2a_ENSTRUT00000004295          --------------------------------------------------
zCRY2a_BAA96848.1                   --------------------------------------------------
zCRY2b_NP_571867.2                  --------------------------------------------------
hCRY2_AAH35161.1                    --------------------------------------------------
cCRY2_NP_989575.1                   --------------------------------------------------
XtCRY2_BAI82612.1                   --------------------------------------------------
TrCRY3_ENSTRUG00000015322           --------------------------------------------------
zCRY3_BAA96850.1                    --------------------------------------------------
Tr[6-4]photolyase_XP_011607950      --------------------------------------------------
z[6-4]photolyase_BAA96852.1         --------------------------------------------------
cCRY4_NP_001034685.1                --------------------------------------------------
XtCRY4_AB534559                     --------------------------------------------------
zCRY4_AAI64413.1                    --------------------------------------------------
DmCRY_NP_732407.1                   --------------------------------------------------
TrCRY-dash_ENSTRUT00000044961       --------------------------------------------------
zCRY-dash_NP_991249.1               --------------------------------------------------
TrCRY6a                             MHSVLTSIYKEYFSTTEDDELELALALSLVETKDYELSAPVHEPQLKRPG 50
AtCry1_AEE82696.1                   --------------------------------------------------
AtCry2_NP_171935.1                  --------------------------------------------------
XlCPDphotolyase_NP_001089127.1      --------------------------------------------------
DmCPDphotolyase_NP_523653.2         --------------------------------------------------


hCRY1_NP_004066.1                   --------------------------------------------------
cCRY1_NP_989576.1                   --------------------------------------------------
XtCRY1_NP_001017311.2               --------------------------------------------------
TrCRY1a_XP_011611545.1              --------------------------------------------------
zCRY1a_CAH69047.1                   --------------------------------------------------
zCRY1b_NP_571865.4                  --------------------------------------------------
TrCRY2a_ENSTRUT00000004295          --------------------------------------------------
zCRY2a_BAA96848.1                   --------------------------------------------------
zCRY2b_NP_571867.2                  --------------------------------------------------
hCRY2_AAH35161.1                    --------------------------------------------------
cCRY2_NP_989575.1                   --------------------------------------------------
XtCRY2_BAI82612.1                   --------------------------------------------------
TrCRY3_ENSTRUG00000015322           --------------------------------------------------
zCRY3_BAA96850.1                    --------------------------------------------------
Tr[6-4]photolyase_XP_011607950      --------------------------------------------------
z[6-4]photolyase_BAA96852.1         --------------------------------------------------
cCRY4_NP_001034685.1                --------------------------------------------------
XtCRY4_AB534559                     --------------------------------------------------
zCRY4_AAI64413.1                    --------------------------------------------------
DmCRY_NP_732407.1                   --------------------------------------------------
TrCRY-dash_ENSTRUT00000044961       --------------------------------------------------
zCRY-dash_NP_991249.1               --------------------------------------------------
TrCRY6a                             GRRNESGSVWPTSEFQTQRRSPIQPAEAASGTRNNSPQTGPSPKVCSPQT 100
AtCry1_AEE82696.1                   --------------------------------------------------
AtCry2_NP_171935.1                  --------------------------------------------------
XlCPDphotolyase_NP_001089127.1      --------------------------------------------------
DmCPDphotolyase_NP_523653.2         --------------------------------------------------


hCRY1_NP_004066.1                   --------------------------------------------------
cCRY1_NP_989576.1                   --------------------------------------------------
XtCRY1_NP_001017311.2               --------------------------------------------------
TrCRY1a_XP_011611545.1              --------------------------------------------------
zCRY1a_CAH69047.1                   --------------------------------------------------
zCRY1b_NP_571865.4                  --------------------------------------------------
TrCRY2a_ENSTRUT00000004295          --------------------------------------------------
zCRY2a_BAA96848.1                   --------------------------------------------------
zCRY2b_NP_571867.2                  --------------------------------------------------
hCRY2_AAH35161.1                    --------------------------------------------------
cCRY2_NP_989575.1                   -------------------------------------------MAAAASP 7
XtCRY2_BAI82612.1                   --------------------------------------------------
TrCRY3_ENSTRUG00000015322           --------------------------------------------------
zCRY3_BAA96850.1                    --------------------------------------------------
Tr[6-4]photolyase_XP_011607950      --------------------------------------------------
z[6-4]photolyase_BAA96852.1         --------------------------------------------------
cCRY4_NP_001034685.1                --------------------------------------------------
XtCRY4_AB534559                     --------------------------------------------------
zCRY4_AAI64413.1                    --------------------------------------------------
DmCRY_NP_732407.1                   --------------------------------------------------
TrCRY-dash_ENSTRUT00000044961       --------------------------------------------------
zCRY-dash_NP_991249.1               --------------------------------------------------
TrCRY6a                             AVEPEHNADAWTPAKARSASCESGHQVMEVGDVDQSEKPKRPKKRRQRRK 150
AtCry1_AEE82696.1                   ---------------------------------------------MSGSV 5
AtCry2_NP_171935.1                  --------------------------------------------------
XlCPDphotolyase_NP_001089127.1      -------------------------------------------------M 1
DmCPDphotolyase_NP_523653.2         -------------------------------------------------M 1


hCRY1_NP_004066.1                   --MGVN------------AVHWFRKGLRLHDNPALKECIQGAD---TIRC 33
cCRY1_NP_989576.1                   --MGVN------------AVHWFRKGLRLHDNPALRECIRGAD---TVRC 33
XtCRY1_NP_001017311.2               --MGVN------------AVHWFRKGLRLHDNPALRECIQGAD---TIRC 33
TrCRY1a_XP_011611545.1              --MVIN------------TIHWFRKGLRLHDNPSLKDSLLGAD---SVRC 33
zCRY1a_CAH69047.1                   --MVVN------------TVHWFRKGLRLHDNPSLRDSILGAH---SVRC 33
zCRY1b_NP_571865.4                  --MVVN------------TVHWFRKGLRLHDNPSLRDSIKGAD---NLRC 33
TrCRY2a_ENSTRUT00000004295          --MAPN------------SIHWFRKGLRLHDNPALREAVRGAG---TVRC 33
zCRY2a_BAA96848.1                   --MAPN------------SIHWFRKGLRLHDNPALQEAVRGAD---TVRC 33
zCRY2b_NP_571867.2                  --MSVN------------SVHWFRKGLRLHDNPALLEALNGAD---TLRC 33
hCRY2_AAH35161.1                    --------------------------------------------------
cCRY2_NP_989575.1                   PRGFCR------------SVHWFRRGLRLHDNPALQAALRGAA---SLRC 42
XtCRY2_BAI82612.1                   ----VS------------SVHWFRKGLRLHDNPALSAALRDAT---SVRC 31
TrCRY3_ENSTRUG00000015322           --MVVN------------SVHWFRKGLRLHDNPALQEALNGAD---SLRC 33
zCRY3_BAA96850.1                    --MVVN------------SVHWFRKGLRLHDNPALQEALNGAD---TVRC 33
Tr[6-4]photolyase_XP_011607950      --MTHT------------CIHWFRKGLRLHDNPGLMAALRDCK---ELYP 33
z[6-4]photolyase_BAA96852.1         --MSHN------------TIHWFRKGLRLHDNPALIAALKDCR---HIYP 33
cCRY4_NP_001034685.1                --MRHR------------TIHLFRKGLRLHDNPALLAALQSSE---VVYP 33
XtCRY4_AB534559                     --MPHR------------TIHIFRKGLRLHDNPTLVTALETSD---VVYP 33
zCRY4_AAI64413.1                    --MSHR------------TIHLFRKGLRLHDNPALLGALASSS---TLYP 33
DmCRY_NP_732407.1                   --MATRGA----------NVIWFRHGLRLHDNPALLAALADKDQGIALIP 38
TrCRY-dash_ENSTRUT00000044961       --MSGPRT----------VICLLRNDLRLFDNELFHWAQRNAD---HIVP 35
zCRY-dash_NP_991249.1               --MSASRT----------VICLLRNDLRLHDNEVFHWAQRNAE---HIIP 35
TrCRY6a                             CAAQQVVGLPRAPSAAAPVLLWFRRDLRLSDNPALVSALKVGAP---VIP 197
AtCry1_AEE82696.1                   SGCGSGGC----------SIVWFRRDLRVEDNPALAAAVRAGP----VIA 41
AtCry2_NP_171935.1                  --MKMDKK----------TIVWFRRDLRIEDNPALAAAAHEGS----VFP 34
XlCPDphotolyase_NP_001089127.1      SHKGKQSKPKDGESGSEKSDGSQKKGMGIDAKTKTQTITKSPGKRKIPGK 51
DmCPDphotolyase_NP_523653.2         FTLASYWR------------ESFKIVLPLQAMKRTAKQAGPSKKAAKNE 39


hCRY1_NP_004066.1                   VYILDP------WFAGSSNVGINRWRFLLQCLEDLDANLRKLN---SRLF 74
cCRY1_NP_989576.1                   VYILDP------WFAGSSNVGINRWRFLLQCLEDLDANLRKLN---SRLF 74
XtCRY1_NP_001017311.2               VYILDP------WFAGSSNVGINRWRFLLQCLEDLDANLRKLN---SRLF 74
TrCRY1a_XP_011611545.1              VYILDP------WFAGSSNVGINRWRFLLQSLEDLDSSLRKLN---SRLF 74
zCRY1a_CAH69047.1                   VYILDP------WFAGSSNVGISRWRFLLQCLEDLDASLRKLN---SRLF 74
zCRY1b_NP_571865.4                  VYILDP------WFAGSSNVGISRWRFLLQCLEDLDASLRKLN---SRLF 74
TrCRY2a_ENSTRUT00000004295          VYFLDP------WFAGSSNVGVNRWRFLLQCLEDLDANLRKLN---SRLF 74
zCRY2a_BAA96848.1                   VYFLDP------WFAGSSNLGVNRWRFLLQCLDDLDSNLRKLN---SRLF 74
zCRY2b_NP_571867.2                  VYFLDP------WFAGASNLGVNRWRFLLQSLEDLDASLRKLN---SCLF 74
hCRY2_AAH35161.1                    --------------------------------------------------
cCRY2_NP_989575.1                   IYILDP------WFAASSAVGINRWRFLLQSLEDLDNSLRKLN---SRLF 83
XtCRY2_BAI82612.1                   VYILDP------WFAASSSGGVNRWRFLLQSLEDLDTSLRKLN---SRLF 72
TrCRY3_ENSTRUG00000015322           IYILDP------WFAGAANVGINRWRFLLEALEDLDCSLRKLS---SRLL 74
zCRY3_BAA96850.1                    VYILDP------WFAGSANVGVNRWRFLLESLEDLDTSLRKLN---SRLS 74
Tr[6-4]photolyase_XP_011607950      VFILDP------QLHNKS-VGVNRCRFLIGALKDLDLSLRQLN---TRLF 73
z[6-4]photolyase_BAA96852.1         LFLLDP------WFPKNTRIGINRWRFLIEALKDLDSSLKKLN---SRLF 74
cCRY4_NP_001034685.1                VYILDRA-----FMTSSMHIGALRWHFLLQSLEDLRSSLRQLG---SCLL 75
XtCRY4_AB534559                     VYILDRN-----FMTSSSVIGSKRWNFFLQSIEDLHCNLQKLN---SCLF 75
zCRY4_AAI64413.1                    VYVLDRV-----FLQGAMHMGALRWRFLLQSLEDLDTRLRAIG---SRLF 75
DmCRY_NP_732407.1                   VFIFDG------ESAGTKNVGYNRMRFLLDSLQDIDDQLQAATDGRGRLL 82
TrCRY-dash_ENSTRUT00000044961       LYCFDPRHYMGTYHYNLPKTGPFRLRFLLESIKDLRNTLLNKG---SNLI 82
zCRY-dash_NP_991249.1               LYCFDPRHYQGTYHYNFPKTGPFRLRFLLDSVKDLRALLKKHG---STLL 82
TrCRY6a                             IFIWSPEE--EEGPGVTVAMGGACKYWLHQALSCLCSSLENIG---SHLV 242
AtCry1_AEE82696.1                   LFVWAP------EEEGHYHPGRVSRWWLKNSLAQLDSSLRSLG---TCLI 82
AtCry2_NP_171935.1                  VFIWCP------EEEGQFYPGRASRWWMKQSLAHLSQSLKALG---SDLT 75
XlCPDphotolyase_NP_001089127.1      VLSD--------SSGDSSGVDAKKKKTMVEQKGEKD----VREAVKISRM 89
DmCPDphotolyase_NP_523653.2         KASSEPK-----SDQESSDEEASTSKALLVSKPDYQNFEQFLTHLEHQRV 84


hCRY1_NP_004066.1                   VIR-------GQPADVFPRLFKEWN-ITKLSIEYDSEP---FGKERDAAI 113
cCRY1_NP_989576.1                   VIR-------GQPADVFPRLFKEWS-IAKLSIEYDSEP---FGKERDAAI 113
XtCRY1_NP_001017311.2               VIR-------GQPADVFPRLFKEWK-ITKLSIEYDSEP---FGKERDAAI 113
TrCRY1a_XP_011611545.1              VIR-------GQPTDVFPRLFKEWN-ISRLSYEYDSEP---FGKERDAAI 113
zCRY1a_CAH69047.1                   VIR-------GQPTDVFPRLFKEWN-INRLSYEYDSEP---FGKERDAAI 113
zCRY1b_NP_571865.4                  VIR-------GQPTDVFPRLFKEWK-ISRLSYEYDSEP---FGKDRDAAI 113
TrCRY2a_ENSTRUT00000004295          VIR-------GQPANVFPRLFKEWK-ISRLTFEYDSEP---FGKERDAAI 113
zCRY2a_BAA96848.1                   VVR-------GQPANVFPRLFKEWK-ISRLTFEYDSEP---FGKERDAAI 113
zCRY2b_NP_571867.2                  VIR-------GQPADIFPRLFKEWK-VSRLTFEFDSEP---FGKERDAAI 113
hCRY2_AAH35161.1                    --------------------------------------------------
cCRY2_NP_989575.1                   VVR-------GQPTDVFPRLFKEWG-VTRLTFEYDSEP---FGKERDAAI 122
XtCRY2_BAI82612.1                   VVR-------GQPADVFPRLFKEWG-VSRLTFEYDSEP---FGKERDAVI 111
TrCRY3_ENSTRUG00000015322           VVR-------GQPTDVFPRLLKDWK-VTRLTFEFDPEP---YGKERDGAI 113
zCRY3_BAA96850.1                    VVR-------GQPTDVFPRLFKEWN-VTRLTFEYDSEP---YGKERDAAI 113
Tr[6-4]photolyase_XP_011607950      VVR-------GKPEEVFPKLFCQWK-ITKLTYEYDTEP---LSLSRDKTV 112
z[6-4]photolyase_BAA96852.1         VVR-------GSPTEVLPKLFKQWK-ITRLTFEVDTEP---YSQSRDKEV 113
cCRY4_NP_001034685.1                VIQ-------GEYESVVRDHVQKWN-ITQVTLDAEMEP---FYKEMEANI 114
XtCRY4_AB534559                     VIQ-------GDYERVLREHVEKWN-ITQVTFDLEIEP---YYKGLDERI 114
zCRY4_AAI64413.1                    VLC-------GSTANILRELVAQWG-ITQISYDTEVEP---YYTRMDKDI 114
DmCRY_NP_732407.1                   VFE-------GEPAYIFRRLHEQVR-LHRICIEQDCEP---IWNERDESI 121
TrCRY-dash_ENSTRUT00000044961       VRR-------GKPEEVVASLIKQLGSVSTVAFHEEVTS---EELDVEKRV 122
zCRY-dash_NP_991249.1               VRQ-------GKPEDVVCELIKQLGSVSTVAFHEEVAS---EEKSVEEKL 122
TrCRY6a                             FLKSETRGSEGGASLRALQGLAKATGARTVVANALYEP---WLKGRDDAV 289
AtCry1_AEE82696.1                   TKR-------STDSVASLLDVVKSTGASQIFFNHLYDP---LSLVRDHRA 122
AtCry2_NP_171935.1                  LIK-------THNTISAILDCIRVTGATKVVFNHLYDP---VSLVRDHTV 115
XlCPDphotolyase_NP_001089127.1      AAA-------PSVSEFKFNKKRARLLSTEADLKEDAQG-IVYWMSRDQRV 131
DmCPDphotolyase_NP_523653.2         CTA-------ANIQEFSFRKKRVRVLSKTEDVKESSLGGVVYWMSRDGRV 127


hCRY1_NP_004066.1                   KK-----LATEAGVEVIVRISHTLYDLDKIIELNGGQPPLTYKRFQTLIS 158
cCRY1_NP_989576.1                   KK-----LASEAGVEVIVRISHTLYDLDKIIELNGGQPPLTYKRFQTLIS 158
XtCRY1_NP_001017311.2               KK-----LASEAGVEVIVRISHTLYDLDKIIELNGGQPPLTYKRFQTLIS 158
TrCRY1a_XP_011611545.1              KK-----LASEAGVEVTVCISHTLYDLDKIIELNGGQSPLTYKRFQTLIS 158
zCRY1a_CAH69047.1                   KK-----LANEAGVEVIVRISHTLYDLDKIIELNGGQSPLTYKRFQTLIS 158
zCRY1b_NP_571865.4                  RK-----LATEAGVEVFVRISHTLYDLDKIIEFNGGQSPLTYKRFQTLIS 158
TrCRY2a_ENSTRUT00000004295          KK-----LAMEAGVEVIVKISHTLYDLDKIIELNGGHPPLTYKRFQTLIS 158
zCRY2a_BAA96848.1                   KK-----LAMEAGVEVIVKTSHTLYNLDKIIELNGGQPPLTYKRFQTLIS 158
zCRY2b_NP_571867.2                  KK-----LACEAGVEVIVKISHTLYDLDRIIELNGGQSPLTYKRFQTLVS 158
hCRY2_AAH35161.1                    MK-----MAKEAGVEVVTENSHTLYDLDRIIELNGQKPPLTYKRFQAIIS 45
cCRY2_NP_989575.1                   IK-----LAKEAGVEVVIENSHTLYDLDRIIELNGNKPPLTYKRFQAIIS 167
XtCRY2_BAI82612.1                   MK-----LAKEAGVEVIENSHTLYDLDRIIELNGHSPPLTYKRFQAIIS 156
TrCRY3_ENSTRUG00000015322           IK-----LAQQFGVETIVRNSHTLYNLDRIIEVNNNSPPLTFKRFQTIVS 158
zCRY3_BAA96850.1                    IK-----MAQEYGVETVVRNSHTLYNPDRIIEMNNHSPPLTFKRFQAIVN 158
Tr[6-4]photolyase_XP_011607950      TR-----LAEEHGIDVVCKVSHTLFDINRIIEENNGKTPLTYKSMQAIVK 157
z[6-4]photolyase_BAA96852.1         MK-----LAKEYGVEVTPKISHTLYNIDRIIDENNGKTPMTYIRLQSVVK 158
cCRY4_NP_001034685.1                RG-----LGEELGFQVLSLMGHSLYNTQRILELNGGTPPLTYKRFLRILS 159
XtCRY4_AB534559                     RA-----MGQELGFEVVSMVAHTLYDIKTILALNCGKPPLTYKNFLRVLS 159
zCRY4_AAI64413.1                    QT-----VAQENGLQTYTCISHTLYDVKRIVKANGGSPPLTYKKFLHVLS 159
DmCRY_NP_732407.1                   RS-----LCRELNIDFVEKVSHTLWDPQLVIETNGGIPPLTYQMFLHTVQ 166
TrCRY-dash_ENSTRUT00000044961       KD-----VCAQMKVNVHTCWGSTLYHRDDLPFHHISRLPDVYTQFRKAVE 167
zCRY-dash_NP_991249.1               KE-----ICCQNKVRVQTFWGSTLYHRDDLPFSHIGGLPDVYTQFRKAVE 167
TrCRY6a                             AA-----ALQKSGVECEMFHSYCLRDPYSVSTEGVGLRGIGSVSHFMSCC 334
AtCry1_AEE82696.1                   KD-----VLTAQGIAVRSFNADLLYEPWEVTDEL-GRPFSMFAAFWERCL 166
AtCry2_NP_171935.1                  KE-----KLVERGISVQSYNGDLLYEPWEIYCEK-GKPFTSFNSYWKKCL 159
XlCPDphotolyase_NP_001089127.1      QDNWAFLYAQRLALKQKLPLHVTFCLVPKFLDATIRHYGFMLKGLQEVAE 181
DmCPDphotolyase_NP_523653.2         QDNWALLFAQRLALKLELPLTVVFCLVPKFLNATIRHYKFMMGGLQEVEQ 177
                                                 .         :     .


hCRY1_NP_004066.1                   KMEPLEIPVETITSE--VIEKCTTPLSDDHDEKYGVPSLEELGFDTDGL- 205
cCRY1_NP_989576.1                   RMEPLEMPVETITPE--VMQKCTTPVSDDHDEKYGVPSLEELGFDTDGL- 205
XtCRY1_NP_001017311.2               KMEPLEIPVETITAE--VMEKCTTPVLDDHDEKYGVPSLEELGFDTEGL- 205
TrCRY1a_XP_011611545.1              RMDPVEVPAESITAE--IMGKCTTPLSDDHDDKYGVPSLEELGFDTEGL- 205
zCRY1a_CAH69047.1                   RMEAVETPAETITAE--VMGPCTTPLSDDHDEKFGVPSLEELGFDTEGL- 205
zCRY1b_NP_571865.4                  RMDPVEMPAETITAE--IMGKCSTPVSDDHDDKFGVPSLEELGFETEGL- 205
TrCRY2a_ENSTRUT00000004295          RMDPPEMPVETLSGN--LMGRCVTPISEDHGEKYGVPSLEELGFDIEGL- 205
zCRY2a_BAA96848.1                   RMDPPEMPVETLSNS--IMGCCVTPVAEDHGDKYGVPSLEELGFDIEGL- 205
zCRY2b_NP_571867.2                  SMEPPDPPLASPDRG--MMGKCVTPISENHRDKYGVPLLEELGFDTEGL- 205
hCRY2_AAH35161.1                    RMELPKKPVGLVTSQ--QMESCRAEIQENHDETYGVPSLEELGFPTEGL- 92
cCRY2_NP_989575.1                   RMELPKKPVSSIVSQ--QMETCKVDIQENHDDVYGVPSLEELGFPTDGL- 214
XtCRY2_BAI82612.1                   RMELPRRPAPSVTRQ--QMEACRAEIKRNHDETYGVPSLDELGFHSEIK- 203
TrCRY3_ENSTRUG00000015322           RLELPRRPLPTVTQH--QIHKCGAKMADSQEQLYSIPSLEELGFRTEGL- 205
zCRY3_BAA96850.1                    RLELPRKPLPTITQE--QMARCRTQISDNHDEHYGVPSLEELGFRTQGD- 205
Tr[6-4]photolyase_XP_011607950      KLGPPKRPLSAPSME--DLKDVNTPCSESHEKKYRIPTLEDFGHNLADL- 204
z[6-4]photolyase_BAA96852.1         AMGHPKKPIPAPTNE--DMRGVSTPLSDDHEEKFGIPTLEDLGLDTSSL- 205
cCRY4_NP_001034685.1                LLGDPEVPVRNPTAE--DFQRCSPP-ELGLAECYGVPLPTDLKIPPE--- 203
XtCRY4_AB534559                     MIGNPDKPARQITSE--DFIKCITPTKLAAEEYYRIPKPEDLGISKD--- 204
zCRY4_AAI64413.1                    VLGEPEKPARDVSIE--DFQRCVTP--VDVDRVYAVPSLADLGLQVE--- 202
DmCRY_NP_732407.1                   IIGLPPRPTADARLEDATFVELDPEFCRSLKLFEQLPTPEHFNVYGDNMG 216
TrCRY-dash_ENSTRUT00000044961       SQCRVRPVFPPPEHLK------PLPQGLEEGTILTAEDLEQKEPVADPR- 210
zCRY-dash_NP_991249.1               AQGRVRPVLSTPEQVK------SPPSGLEEGPIPTFDSLGQTEPLDDCR- 210
TrCRY6a                             RQNPGAALGAPLDPPTTLPVPAHWPPGVSLETLGLARMPKRKDGTTIDWA 384
AtCry1_AEE82696.1                   SMPYD----PESPLLP-PKKIISGDVSKCVADPLVFEDDSEKGSNALLA- 210
AtCry2_NP_171935.1                  DMSIESVMLPPPWRLM-PITAAEAVWACSIEELGLENEAEKPSNALLT- 207
XlCPDphotolyase_NP_001089127.1      ECKELNIPFHLLIGY---AKDILPNFVKDHAIGGVVTDFSPLRVPLQWV- 227
DmCPDphotolyase_NP_523653.2         QCRALDIPFHLLMGS---AVEKLPQFVKSKDIGAVVCDFAPLRLPRQWV- 223


hCRY1_NP_004066.1                   --SSAVWPGGETEALTRLERHLER---KAWVANFERPRMNANSLLASPTG 250
cCRY1_NP_989576.1                   --PSAVWPGGETEALTRLERHLER---KAWVANFERPRMNANSLLASPTG 250
XtCRY1_NP_001017311.2               --PSAVWPGGETEALTRLERHLER---KAWVANFERPRMNANSLLASTTG 250
TrCRY1a_XP_011611545.1              --SSAVWPXGETEALTRLERHLER---KAWVANFERPRMNANSLLASPTG 250
zCRY1a_CAH69047.1                   --SSAVWPGGETEALTRLERHLER---KAWVANFERPRMNANSLLASPTG 250
zCRY1b_NP_571865.4                  --STAVWPGGETEALTRLERHLER---KAWVANFERPRMNANSLLASPTG 250
TrCRY2a_ENSTRUT00000004295          --PSAVWPGGETEALTRIERHLER---KAWVANFERPRMNANSLLASPTG 250
zCRY2a_BAA96848.1                   --PSAVWPGGETEALTRIERHLER---KAWVANFERPRMNANSLLASPTG 250
zCRY2b_NP_571867.2                  --APAVWPGGESEALKRMERHLGPDSTVAWQENFERPKMNASPLMASPLG 253
hCRY2_AAH35161.1                    --GPAVWQGGETEALARLDKHLER---KAWVANYERPRMNANSLLASPTG 137
cCRY2_NP_989575.1                   --APAVWQGGETEALARLDKHLER---KAWVANYERPRMNANSLLASPTG 259
XtCRY2_BAI82612.1                   --GPSIWPGGETEALARLDRHLER---KAWVANYERPRMNANSLLASPTG 248
TrCRY3_ENSTRUG00000015322           --PPAVWRGGESEALERLHKHLDK---KVWVANLEHSRVSTCSLYASPAG 250
zCRY3_BAA96850.1                    --SLHVWKGGETEALERLNKHLDR---KAWVANYERPRISGQSLFPSPTG 250
Tr[6-4]photolyase_XP_011607950      --PEEQFPGGEQEALRRLEEHMKR---TAWVCNFEKPKTSPNSLSPSTTV 249
z[6-4]photolyase_BAA96852.1         --GPHLFPGGEQEALRRLDEHMER---TNWVCKFEKPKTSPNSLIPSTTV 250
cCRY4_NP_001034685.1                --SISPWRGGESEGLQRLEQHLAD---QGWVASFTKPKTVPNSLLPSTTG 248
XtCRY4_AB534559                     --CPTNWVGGESEALSRLEQHLEK---QGWVANFKKPQTIPNSLLPSTTG 249
zCRY4_AAI64413.1                    --AEVLWPGGESHALQRLEKHFQS---QGWVANFSKPRTIPNSLLPSTTG 247
DmCRY_NP_732407.1                   FLAKINWRGGETQALLLLDERLKVEQ-HAFERGFYLPNQALPNIHDSPKS 265
TrCRY-dash_ENSTRUT00000044961       --SAFPCSGGESQALARLKHYFWD---TDAVAVYKETRNGLIGVDYS-TK 254
zCRY-dash_NP_991249.1               --SAFPCRGGETEALARLKHYFWD---TNAVATYKETRNGMIGVDFS-TK 254
TrCRY6a                             ANIRKSWDFSEGGAHARLEAFLHDG-------VYRYEKESGRADAPNTSC 427
AtCry1_AEE82696.1                   ----RAWSPGWSNGDKALTTFING-------PLLEYSKNRRKADSATTSF 249
AtCry2_NP_171935.1                  ----RAWSPGWSNADKLLNEFIEK-------QLIDYAKNSKKVVGNSTSL 246
XlCPDphotolyase_NP_001089127.1      --EDVGKCLPEDVPLVQVDAHNIVP---CWVASIKQEYGARTIRRKVHDQ 272
DmCPDphotolyase_NP_523653.2         --EDVGKALPKSVPLVQVDAHNVVP---LWVASDKQEYAARTIRNKINSK 268


hCRY1_NP_004066.1                   LSPYLRFGCLSCRLFYFKLTDLYKKVKKNSSPP-------LSLYGQLLWR 293
cCRY1_NP_989576.1                   LSPYLRFGCLSCRLFYFKLTDLYKKVKKNSSPP-------LSLYGQLLWR 293
XtCRY1_NP_001017311.2               LSPYLRFGCLSCRLFYFKLTDLYKKVKKNSSPP-------LSLYGQLLWR 293
TrCRY1a_XP_011611545.1              LSPYLRFGCLSCRLFYFKLTDLYRKVKKNSSPP-------LSLYGQLLWR 293
zCRY1a_CAH69047.1                   LSPYLRFGCLSCRLFYFKLTDLYRKVKKNSSPP-------LSLYGQLLWR 293
zCRY1b_NP_571865.4                  LSPYLRFGCLSCRLFYFKLTDLYRKVKKNSTPS-------LSLYGQLLWR 293
TrCRY2a_ENSTRUT00000004295          LSPYLRFGCLSCRLFYFKLTDLYRKVKKNSSPP-------LSLYGQLLWR 293
zCRY2a_BAA96848.1                   LSPYLRFGCLSCRLFYFKLTDLYRKVKKTSTPP-------LSLYGQLLWR 293
zCRY2b_NP_571867.2                  LSPYLRFGCLSCRLFYCKLTQLYKKVKKNMNPS-------ISLYDKILWR 296
hCRY2_AAH35161.1                    LSPYLRFGCLSCRLFYYRLWDLYKKVKRNSTPP-------LSLFGQLLWR 180
cCRY2_NP_989575.1                   LSPYLRFGCLSCRLFYYRLWELYKKVKRNSTPP-------LSLYGQLLWR 302
XtCRY2_BAI82612.1                   LSPYLRFGCLSCRLFYYRLKELYKKVKKNSPPP-------LSLYGQLLWR 291
TrCRY3_ENSTRUG00000015322           LSPYLRFGCLSCRVLYYNLRELYVKLRKGCSPP-------PSLFGQLLWR 293
zCRY3_BAA96850.1                    LSPYLRFGCLSCRVFYYNLRDLFMKLRRRSSPP-------LSLFGQLLWR 293
Tr[6-4]photolyase_XP_011607950      LSPYVTFGCLSVRTFWWRLSDVYE-GKKHSAPP-------VSLHGQLLWR 291
z[6-4]photolyase_BAA96852.1         LSPYVRFGCLSARTFWWRLADVYR-GKTHSDPP-------VSLHGQLLWR 292
cCRY4_NP_001034685.1                LSPYFSTGCLSVRSFFYRLSNIYAQAKHHSLPP-------VSLQGQLLWR 291
XtCRY4_AB534559                     LSPYFSLGCLSVRVFFHRLSNIYAQSKNHSLPP-------VSLQGQLLWR 292
zCRY4_AAI64413.1                    LSPYLSLGCLSVRTFYHRLNSIYAQSKNHSLPP-------VSLQGQVLWR 290
DmCRY_NP_732407.1                   MSAHLRFGCLSVRRFYWSVHDLFKNVQLRACVRGVQMTGGAHITGQLIWR 315
TrCRY-dash_ENSTRUT00000044961       FSPWLALGCISPRYIYHQIKQYESERTANQSTY--------WVIFELLWR 296
zCRY-dash_NP_991249.1               FSPWLALGCISPRYIYEQIKKYEVERTANQSTY--------WVIFELLWR 296
TrCRY6a                             VSPYLHFGQLSPRWLLWDAKG------ARCRPP--------KFQRKLAWR 463
AtCry1_AEE82696.1                   LSPHLHFGEVSVRKVFHLVRIKQVAWANEGNEAGEE--SVNLFLKSIGLR 297
AtCry2_NP_171935.1                  LSPYLHFGEISVRHVFQCARMKQIIWARDKNSEGEE--SADLFLRGIGLR 294
XlCPDphotolyase_NP_001089127.1      LSQFLTEFPPVIKHPYEPKFE-AETVDWDKCYK--------SLEVDRTVG 313
DmCPDphotolyase_NP_523653.2         LGEYLSVFPPVVRHPHGTGCKNVNTVDWSSAAYA--------SLQCDMEVD 310
                                    .. .       :                             .


hCRY1_NP_004066.1                   EFFYTAATNNPRFDKMEGNPICVQIPWDK-NPEALAKWAEGRTGFPWIDA 342
cCRY1_NP_989576.1                   EFFYTAATNNPRFDKMEGNPICVQIPWDK-NPEALAKWAEGRTGFPWIDA 342
XtCRY1_NP_001017311.2               EFFYTAATNNPRFDKMEGNPICVQIPWDR-NPEALAKWAEGRTGFPWIDA 342
TrCRY1a_XP_011611545.1              EFFYTAATNNPCFDKMENNPICVQIPWDR-NPEALAKWAEGRTGFPWIDA 342
zCRY1a_CAH69047.1                   EFFYTAATNNPRFDKMEGNPICVQIPWDK-NPEALAKWAEGRTGFPWIDA 342
zCRY1b_NP_571865.4                  EFFYTAATNNPHFDKMEFNPICVQIPWDR-NPEALAKWAEGQTGFPWIDA 342
TrCRY2a_ENSTRUT00000004295          EFFYTAATNNPRFDKMEGNPICVRIPWDR-NMEALAKWAEAKTGFPWIDA 342
zCRY2a_BAA96848.1                   EFFYTAATTNPRFDKMEGNPICVRIPWDK-NPEALAKWAEAKTGFPWIDA 342
zCRY2b_NP_571867.2                  EFFYTAATNNPRFDRMEGNPICIRIPWDR-NAEALAKWAEAKTGFPWIDA 345
hCRY2_AAH35161.1                    EFFYTAATNNPRFDRMEGNPICIQIPWDR-NPEALAKWAEGKTGFPWIDA 229
cCRY2_NP_989575.1                   EFFYTAATNNPKFDRMEGNPICIQIPWDK-NPEALAKWAEGKTGFPWIDA 351
XtCRY2_BAI82612.1                   EFFYTAATNNPKFDQMEGNPICVQIPWDK-NPKALAKWAEGKTGFPWIDA 340
TrCRY3_ENSTRUG00000015322           EFFYTAATNNPNFDRMEGNPICVQIPWDQ-NPEALAKWAEGHTGFPWIDA 342
zCRY3_BAA96850.1                    EFFYTAGTNNPNFDHMEGNPICVQIPWDH-NPEALAKWAEGRTGFPWIDA 342
Tr[6-4]photolyase_XP_011607950      EFFYTASVGISNFNKMVDNPVCTQVDWDI-NSEYLAAWREARTGFPFIDA 340
z[6-4]photolyase_BAA96852.1         EFFYTTAVGIPNFNKMEGNSACVQVDWDN-NPEHLAAWREARTGFPFIDT 341
cCRY4_NP_001034685.1                EFFYTVASATPNFTKMAGNPICLQIRWYE-DAERLHKWKTAQTGFPWIDA 340
XtCRY4_AB534559                     EFFYTVASSTPNFTHMVGNPICLQIDWYK-NEEQLQKWKEAKTGFPWIDA 341
zCRY4_AAI64413.1                    EFFYTVASATPNFTKMEGNSICLQIDWYH-DPERLEKWRTAQTGFPWIDA 339
DmCRY_NP_732407.1                   EYFYTMSVNNPNYDRMEGNDICLSIPWAKPNENLLQSWRLGQTGFPLIDG 365
TrCRY-dash_ENSTRUT00000044961       DYFRFVAVKYGTKLFQVNGLQDKSVSWRK-DMKLFNAWKEGKTGVPFVDA 345
zCRY-dash_NP_991249.1               DYFKFVALKYGNRIFYMNGLQDKHVPWKT-DMKMFDAWKEGRTGVPFVDA 345
TrCRY6a                             DLAYWQLTLFPDLPWESLRPPYKALRWST-DRRHLEAWQRGRTGYPLVDA 512
AtCry1_AEE82696.1                   EYSRYISFNHPYSHERPLLGHLKFFPWAV-DENYFKAWRQGRTGYPLVDA 346
AtCry2_NP_171935.1                  EYSRYICFNFPFTHEQSLLSHLRFFPWDA-DVDKFKAWRQGRTGYPLVDA 343
XlCPDphotolyase_NP_001089127.1      EVEWAKPGTNAGFNVLQSFISERLKHFNSDRNNPNQNALSNLSPWLHFGQ 363
DmCPDphotolyase_NP_523653.2         EVQWAKPGYKAACQQLYEFCSRRLRHFNDKRNDPTADALSGLSPWLHFGH 360
                                    :                         :               :    ..


hCRY1_NP_004066.1                   IMTQLRQEGWIHHLARHAVACFLTRGDLWISWEEGMKVFEELLLDADWSI 392
cCRY1_NP_989576.1                   IMTQLRQEGWIHHLARHAVACFLTRGDLWISWEEGMKVFEELLLDADWSV 392
XtCRY1_NP_001017311.2               IMTQLRQEGWIHHLARHAVACFLTRGDLWISWEEGMKVFEELLLDADWSV 392
TrCRY1a_XP_011611545.1              IMTQLRQEGWIHHLARHAVACFLTRGDLWIGWEEGMKVFEELLLDADWSV 392
zCRY1a_CAH69047.1                   IMTQLRQEGWIHHLARHAVACFLTRGDLWISWEEGMKVFEELLLDADWSV 392
zCRY1b_NP_571865.4                  IMTQLRQEGWIHHLARHAVACFLTRGDLWISWEEGMKVFEELLLDADWSV 392
TrCRY2a_ENSTRUT00000004295          IMTQLRQEGWIHHLARHAVACFLTRGDLWISWEEGMKVFDELLLDADWSV 392
zCRY2a_BAA96848.1                   IMTQLRQEGWIHHLARHAVACFLTRGDLWISWEEGMKVFEELLLDADWSV 392
zCRY2b_NP_571867.2                  IMMQLRQEGWIHHLARHAVACFLTRGDLWISWEEGMKVFEELLLDADWSV 395
hCRY2_AAH35161.1                    IMTQLRQEGWIHHLARHAVACFLTRGDLWVSWESGVRVFDELLLDADFSV 279
cCRY2_NP_989575.1                   IMTQLRQEGWIHHLARHAVACFLTRGDLWISWESGVRVFDELLLDADFSV 401
XtCRY2_BAI82612.1                   IMTQLRQEGWIHHLARHAVACFLTRGDLWNSWECGVKVFDELLLDADFSV 390
TrCRY3_ENSTRUG00000015322           IMTQLRQEGWIHHQARRAVACFLTRGDLWISWECGMKVFEELLLDADWSV 392
zCRY3_BAA96850.1                    IMTQLRQEGWIHHLARHAVACFLTRGDLWISWEEGMKVFEELLLDADWSV 392
Tr[6-4]photolyase_XP_011607950      VMTQLRQQGWIHHLARHAVACFLTRGDLWISWEEGQKVFEELLLDGDWAL 390
z[6-4]photolyase_BAA96852.1         IMTQLRQEGWIHHLARHAVACFLTRGDLWISWEEGQKVFEELLLDSDWSL 391
cCRY4_NP_001034685.1                IMTQLRQEGWIHHLARHAAACFLTRGDLWISWEEGMKVFEELLLDADYSI 390
XtCRY4_AB534559                     IMTQLHNEGWIHHLARHAVACFLTRGDLWISWEEGMKVFEEFLLDADYCI 391
zCRY4_AAI64413.1                    IMTQLRQEGWIHHLARHAVACFLTRGDLWITWEEGMKVFEEFLLDADYSV 389
DmCRY_NP_732407.1                   AMRQLLAEGWLHHTLRNTVATFLTRGGLWQSWEHGLQHFLKYLLDADWSV 415
TrCRY-dash_ENSTRUT00000044961       NMRELATTGFMSNRGRQNVASFLTK-DLGLDWRMGAEWFEYLLVDHDVCS 394
zCRY-dash_NP_991249.1               NMRELALTGFMSNRGRQNVASFLTK-DLGLDWRLGAEWFEYLLVDHDVCS 394
TrCRY6a                             AMRQLWLTGWMNNYMRHVVASFLIA-YLHLPWQEGYRWFQDTLVDADVAI 561
AtCry1_AEE82696.1                   GMRELWATGWLHDRIRVVVSSFFVK-VLQLPWRWGMKYFWDTLLDADLES 395
AtCry2_NP_171935.1                  GMRELWATGWMHNRIRVIVSSFAVK-FLLLPWKWGMKYFWDTLLDADLEC 392
XlCPDphotolyase_NP_001089127.1      LSVQRAILEVQKYRSKFKESVDSFVEEAVVRRELA-DNFCFYNKNYDKIE 412
DmCPDphotolyase_NP_523653.2         ISAQRCALEVQRFRGQHKASADAFCEEAIVRRELA-DNFCFYNEHYDSLK 409
                                       :           :   :            . .   *     . *


hCRY1_NP_004066.1                   NAGSWMWLSCSSFFQQFFH--CYCPVGFGRRTDPNGDYIRRYLPVLRGFP 440
cCRY1_NP_989576.1                   NAGSWMWLSCSSFFQQFFH--CYCPVGFGRRTDPNGDYIRRYLPVLRGFP 440
XtCRY1_NP_001017311.2               NAGSWMWLSCSSFFQQFFH--CYCPVGFGRRTDPNGDYIRRYLPILKGFP 440
TrCRY1a_XP_011611545.1              NAGSWMWLSCSSFFQQFFH--CYCPVGFGRRTDPNGDYIRRYLPILRGFP 440
zCRY1a_CAH69047.1                   NAGSWMWLSCSSFFQQFFH--CYCPVSFGRRTDPNGDYIRRYLPVLRGFP 440
zCRY1b_NP_571865.4                  NAGSWMWLSCSSFFQQFFH--CYCPVGFGRRTDPNGDYIRRYLPILRGFP 440
TrCRY2a_ENSTRUT00000004295          NAGSWMWLSCSSFFQQFFH--CYCPVGFGRRTDPNGDFIRRYLPILRGFP 440
zCRY2a_BAA96848.1                   NAGSWMWLSCSSFFQQFFH--CYCPVGFGRRTDPNGDFIRRYLPILRGFP 440
zCRY2b_NP_571867.2                  NAGSWLCHSCSSFFQQFFH--CYCPVGFGRRIDPNGDFIRRYLPVLRDFP 443
hCRY2_AAH35161.1                    NAGSWMWLSCGAFFQQFFH--CYCPVGFGRRTDPSGDYIRRYLPKLKAFP 327
cCRY2_NP_989575.1                   NAGSWMWLSCSAFFQQFFH--CYCPVGFGRRTDPSGDYVKRYLPVLKGFP 449
XtCRY2_BAI82612.1                   NAGSWMWLSCSAFFQQFFH--CYCPVGFGRRTDPSGDYVRRYLPVLKAFP 438
TrCRY3_ENSTRUG00000015322           NAGSWMWLSCSAFFQQFFK--CYCPVGFGRRTDPSGDYIRRYIPILKDYP 440
zCRY3_BAA96850.1                    NAGSWMWLSCSAFFQQFFH--CYCPVGFGRRTDPSGDYIRRYIPKLKDYP 440
Tr[6-4]photolyase_XP_011607950      NAGNWQWLSASAFFHQFFR--VYSPVAFGKKTDKNGDYIKKFLPHLKKFP 438
z[6-4]photolyase_BAA96852.1         NAGNWQWLSASTFFHQYFR--VYSPIAFGKKTDKHGDYIKKYLPVLKKFP 439
cCRY4_NP_001034685.1                NAGNWMWLSASAFFHHYTR--IFCPVRFGRRTDPEGQYIRKYLPILKNFP 438
XtCRY4_AB534559                     NAGNWMWLSASAFFHHYTR--IFCPVRFGRRSDPEGNYIRKYLPVLKNFP 439
zCRY4_AAI64413.1                    NAGNWMWVSASAFFHKYTR--IFCPVRFGRRTDPQGEYLRKYLPVLKNFP 437
DmCRY_NP_732407.1                   CAGNWMWVSSSAFERLLDSSLVTCPVALAKRLDPDGTYIKQYVPELMNVP 465
TrCRY-dash_ENSTRUT00000044961       NYGNWLYSAGIGNDPRENR--KFNMIKQGLDYDNNGEYVRLWVPELQRIM 442
zCRY-dash_NP_991249.1               NYGNWLYSAGIGNDPRENR--KFNMIKQGLDYDNNGDYVRQWVPELRGIK 442
TrCRY6a                             DAMMWQNGGMCGLDHWNFV---MHPVDAAMTCDPCGQYVRKWCPELSGLP 608
AtCry1_AEE82696.1                   DALGWQYITGTLPDSREFDR-IDNPQFEGYKFDPNGEYVRRWLPELSRLP 444
AtCry2_NP_171935.1                  DILGWQYISGSIPDGHELDR-LDNPALQGAKYDPEGEYIRQWLPELARLP 441
XlCPDphotolyase_NP_001089127.1      GAYDWAKNTLKDHAKDKRTH-LYTLEKLEAGKTHDPLWNAAQLQMVHEGK 461
DmCPDphotolyase_NP_523653.2         GLSSWAYQTLDAHRKDKRDP-CYSLEELEKSLTYDDLWNSAQLQLVREGK 458
                                        *                                :       :


hCRY1_NP_004066.1                   AKYIYDPWNAPEGIQKVAKCLIGVNYPKPMVNHAEASR-LNIERMKQIYQ 489
cCRY1_NP_989576.1                   AKYIYDPWNAPESVQKAAKCVIGVNYPKPMVNHAEASR-LNIERMKQIYQ 489
XtCRY1_NP_001017311.2               PKYIYDPWNAPETVQKVAKCIIGVNYPKPMVNHAEASR-LNIERMKQIYQ 489
TrCRY1a_XP_011611545.1              AKYIYDPWNAPESVQKAAKCMIGVHYPKPMVNHAEASR-LNIERMKQIYQ 489
zCRY1a_CAH69047.1                   AKYIYDPWNAPESVQKAAKCIIGVHYPMPMVHHAEASR-LNIERMKQIYQ 489
zCRY1b_NP_571865.4                  AKFIYDPWNAPESVQKVAKCIIGVHYPKPMVNHAEASR-INIERMKQIYQ 489
TrCRY2a_ENSTRUT00000004295          AKYIYDPWNAPEAVQQAAKCVIGVHYPKPMVNHAEASR-LNIERMKQIYQ 489
zCRY2a_BAA96848.1                   AKYIYDPWNAPDSVQAAAKCIIGVHYPKPMVNHAEASR-LNIERMKQIYQ 489
zCRY2b_NP_571867.2                  AKYIYDPWNAPHDVQLAAKCVIGVDYPKPMVNHAEASR-LNIERMRQIYQ 492
hCRY2_AAH35161.1                    SRYIYEPWNAPESIQKAAKCIIGVDYPRPIVNHAETSR-LNIERMKQIYQ 376
cCRY2_NP_989575.1                   SRYIYEPWNAPESVQKAAKCIIGVDYPKPMVNHAETSR-LNIERMKQIYQ 498
XtCRY2_BAI82612.1                   SRYIYEPWSAPESVQKEAKCIIGIDYPKPIVNHAEASR-MNIERMKQTYQ 487
TrCRY3_ENSTRUG00000015322           NRYIYEPWNAPEAVQKAANCVVGVDYPRPMINHAEGSR-LNIERMKQVYQ 489
zCRY3_BAA96850.1                    NRYIYEPWNAPESVQKAANCIVGVDYPKPMINHAESSR-LNIERMKQVYQ 489
Tr[6-4]photolyase_XP_011607950      AEYIFEPWKAPQSVQQAAGCIVGKDYPHPIVQHEVVSK-KNIQRMKAAYA 487
z[6-4]photolyase_BAA96852.1         TEYIYEPWKAPRSVQERAGCIVGKDYPRPIVDHEVVHK-KNILRMKAAYA 488
cCRY4_NP_001034685.1                SKYIYEPWTASEEEQKQAGCIIGRDYPFPMVDHKEASD-HNLQLMKQARE 487
XtCRY4_AB534559                     AKYIYEPWTAPEEIQKQAGCLIGKDYPLPMVDHKTASE-HNLQLMRQVRE 488
zCRY4_AAI64413.1                    SQYIYEPWKAPEDVQLSAGCIIGKDYPRPIVSHIEASQ-RNLALMRQVRT 486
DmCRY_NP_732407.1                   KEFVHEPWRMSAEQQEQYECLIGVHYPERIIDLSMAVK-RNMLAMKSLRN 514
TrCRY-dash_ENSTRUT00000044961       GADVHTPWTLSSAILSHAHLSLGETYPTPIIVAPEWSR-HFNKKMSSGAGP 491
zCRY-dash_NP_991249.1               GGDVHTPWTLSNSALSHAQVSLNQTYPCPIITAPEWSR-HVNNKSSGP-S 490
TrCRY6a                             DELIHKPWRCPTSLLRRAGVVFGQTYPERIVTDLEERRSQSLQDVALVRK 658
AtCry1_AEE82696.1                   TDWIHHPWNAPESVLQAAGIELGSNYPLPIVGLDEAKA-RLHEALSQMWQ 493
AtCry2_NP_171935.1                  TEWIHHPWDAPLTVLKASGVELGTNYAKPIVDIDTARE-LLAKAISRTRE 490
XlCPDphotolyase_NP_001089127.1      MHGFLRMYWAKKILEWTSSPEEALHFSLYLNDRYELDG-RDPNGYVGCMW 510
DmCPDphotolyase_NP_523653.2         MHGFLRMYWAKKILEWTATPEHALEYAILLNDKYSLDG-RDPNGYVGCMW 507
                                       .   :                 :.  :


hCRY1_NP_004066.1                   QLSRYRGLGLLASVPSNPNGN--------------------GGFMGYS-A 518
cCRY1_NP_989576.1                   QLSRYRGLGLLATVPSNPNGNGN------------------GGLMSFSPG 521
XtCRY1_NP_001017311.2               QLSRYRGLGLLASVPSNPNGNGN------------------GGLMSYSPG 521
TrCRY1a_XP_011611545.1              QLSCYRGLGLLATVPSNSNGN-RN----------------SNNETCFPV- 521
zCRY1a_CAH69047.1                   QLSCYRGLGLLAMVPSNPNGNGEN----------------STSLMGFKTG 523
zCRY1b_NP_571865.4                  QLSCYRGLGLLATVPSNTANN-ED----------------SNG--AGPSS 520
TrCRY2a_ENSTRUT00000004295          QLSRYRGLGLLASVPSTNGNGSGGLMAYSPGEQQQLPGPNSSTNSHLPGL 539
zCRY2a_BAA96848.1                   QLSRYRGLGLLASVPSTH-NGNGNGMAYSPGEQQ------SGTNTPAPAV 532
zCRY2b_NP_571867.2                  QLSRYRGLSLLATVPSNHIEDAAAN----------------VNRGAMAPP 526
hCRY2_AAH35161.1                    QLSRYRGLCLLASVPSCVEDLSHP-----------------VAEPSSSQA 409
cCRY2_NP_989575.1                   QLSRYRGLCLLASVPSCVEDLSGP-----------------VTDSAPGQG 531
XtCRY2_BAI82612.1                   QLSRYRGLCILASVPSCMEDLGGP-----------------MADSSQ--- 517
TrCRY3_ENSTRUG00000015322           QLSHYRGLSLLASVPTIQEEAEPP-----------------MTDEFQNSS 522
zCRY3_BAA96850.1                    QLSHYRGLSLLASVPTIQEEAEPP-----------------MSDDSQASS 522
Tr[6-4]photolyase_XP_011607950      KRSAN-------------------------------------TAKSLSKI 500
z[6-4]photolyase_BAA96852.1         KRSP--------------------------------------EDKTINKG 500
cCRY4_NP_001034685.1                EQHRIAQLTRDDADDPMEMKLKR-------------------DHSEESFT 518
XtCRY4_AB534559                     EQQKTAQLTKDIADDPMELNLKHPF----------------KNDKECGLE 522
zCRY4_AAI64413.1                    EQQTTAELTRDVADDPMEAGLKR------------------ELREEEGLL 518
DmCRY_NP_732407.1                   SLIT--------------------------------------PPPHCRPS 526
TrCRY-dash_ENSTRUT00000044961       SPRGRKG--------------------------------------PSHTP 503
zCRY-dash_NP_991249.1               SSKGRKG--------------------------------------SSYTA 502
TrCRY6a                             EFGQYVDKRSGCDLVPLPPRLVSEALG--------------SSHLDGGVA 694
AtCry1_AEE82696.1                   LEAASRAAIENGSEEGLGDSAEVEEAP-----------IEFPRDITMEET 532
AtCry2_NP_171935.1                  AQIMIGAAPD-----------EIVADS-----------FEALGANTIKEP 518
XlCPDphotolyase_NP_001089127.1      SICGIHDQG-------------------------------WAERAVFGKI 529
DmCPDphotolyase_NP_523653.2         SIGGVHDMG-------------------------------WKERAIFGKV 526


hCRY1_NP_004066.1                   ENIPGCSSSG---------------SCS---------------------- 531
cCRY1_NP_989576.1                   ESISGCSSAGGAQLGTGDGQTVGVQTCALGDSHTGGS-----GVQQQGYC 566
XtCRY1_NP_001017311.2               ESMSGCSNNGGGQMGVNEGSSASNPNANKGEVHPGTS-----GLQ--GYW 564
TrCRY1a_XP_011611545.1              EASHDPSTPSGYQVPVHTQGDWQSGVMMYMQ-DPQSG-----MGAHQQGY 565
zCRY1a_CAH69047.1                   DMTKEVTTPSGYQMPPTSQGEWHGRTMVYSQGDQQTS-----SIMTSQGF 568
zCRY1b_NP_571865.4                  GDTQERTHITVHTLPASSQSEFPAFAVSCGQ--QLVN-----SVQPQPGY 563
TrCRY2a_ENSTRUT00000004295          SGGSVATGNGSGSILLNFDNEEPARSIGAGQQRQPPSPLPPPQPQQPGYQ 589
zCRY2a_BAA96848.1                   SSGSVASGNRSGSILLNFDSEEHQGPSGIQQQHQQQ------QQQQLGYH 576
zCRY2b_NP_571867.2                  TRRKTRNDASSYRVRREQPGPSGAKHRHRHSLSHRPS----------PYA 566
hCRY2_AAH35161.1                    GSMSSAGPRPLPSGPASPKRKLEAAEEPPGEELS---------------- 443
cCRY2_NP_989575.1                   SSTSTAVRLPQ-SDQASPKRKHEGAEELCTEELY---------------- 564
XtCRY2_BAI82612.1                   -NISEAAPKQSLCNVESPKRKRKVSEEEP--------------------- 545
TrCRY3_ENSTRUG00000015322           GPGRRAGSDPRTAPRSPIPPP----------------------------- 543
zCRY3_BAA96850.1                    SSTGQASSPPHLSITAPSTPPLSESSSPNSSPTASTS------------V 560
Tr[6-4]photolyase_XP_011607950      QGLKRKPSSSVDMLKKKKKNNTSEPLL----------------------- 527
z[6-4]photolyase_BAA96852.1         E--KRKASPSIKEMFQKKAKR----------------------------- 519
cCRY4_NP_001034685.1                KTKAAR-----MTEQT---------------------------------- 529
XtCRY4_AB534559                     NNKVKRRCLEEMTAEDGTRQSDLVQSLDPCEVKVC--------------- 557
zCRY4_AAI64413.1                    EEAESQCTSKRFSGSSDHKSRPCSWTPETLQLSELSGEVM---------- 558
DmCRY_NP_732407.1                   NEEEVRQFFWLADVVV---------------------------------- 542
TrCRY-dash_ENSTRUT00000044961       KQHRDRGIDFYFSRSKNL-------------------------------- 521
zCRY-dash_NP_991249.1               RQHKDRGIDFYFSKNKHF-------------------------------- 520
TrCRY6a                             TGGKQFLLPVITRMEFKHQQEDPDADAASNPYNAVLKGYVSRKRDETIAF 744
AtCry1_AEE82696.1                   EPTRLNPNRRYEDQMVPSIT----SSLIRPEEDEESSLNLRNSVGDSRAE 578
AtCry2_NP_171935.1                  G---LCPSVSSNDQQVPSAVRYNGSKRVKPEEEEE--------------- 550
XlCPDphotolyase_NP_001089127.1      RYMNYQGCKRKFDVAQFERRYHPKKFSQ---------------------- 557
DmCPDphotolyase_NP_523653.2         RYMNYQGCRRKFDVNAFVMRYGGKVHKKK--------------------- 555


hCRY1_NP_004066.1                   QGSGILHYAHGDSQQTHLLKQGRSSMGTG---LSGGKRPSQEEDTQSIG- 577
cCRY1_NP_989576.1                   QASSILRYAHGDNQQSHLMQPGRASLGTG---ISAGKRPNPEEETQSVG- 612
XtCRY1_NP_001017311.2               QGSSILHYSHSDSQQSYLMQ-ARNPLHSV---VSSGKRPNPEEETQSIG- 609
TrCRY1a_XP_011611545.1              AGTSSGVMCYTQNTQQIPGPSTQKGLERHGGIQTTGKRHN-EDSGNGKS- 613
zCRY1a_CAH69047.1                   GNNGS-TMCYRQDAQQITG----RGLHSS-IIQTSGKRHS-EESGPTTV- 610
zCRY1b_NP_571865.4                  SGAGMVGQAHKQ-TQRVRG-------------MTAFKRHG-EDRTTDFP- 597
TrCRY2a_ENSTRUT00000004295          SAPEPSQNVTSSLLYHEFAVPQHPGLLLHTRG-ITGKRERESERDGAGEK 638
zCRY2a_BAA96848.1                   HMPDSGHN--SRFYKSNVSHDMAAGHLLHKGGSVTGKRERESERDLDGEE 624
zCRY2b_NP_571867.2                  TQSELTDHNHEFAVPQQPGRVSWPNVG------GTGKKEGEAEHNCCRGD 610
hCRY2_AAH35161.1                    KRARVAELPTPELPSKDA-------------------------------- 461
cCRY2_NP_989575.1                   KRAKVTGLPAPEIPGKSS-------------------------------- 582
XtCRY2_BAI82612.1                   -HMKVRVLCAPET------------------------------------- 557
TrCRY3_ENSTRUG00000015322           --------------------------------------------------
zCRY3_BAA96850.1                    PHTQRKRVRPSETPSKQKTKVKHTSQARGLELKMDDKQ------------ 598
Tr[6-4]photolyase_XP_011607950      --------------------------------------------------
z[6-4]photolyase_BAA96852.1         --------------------------------------------------
cCRY4_NP_001034685.1                --------------------------------------------------
XtCRY4_AB534559                     --------------------------------------------------
zCRY4_AAI64413.1                    --------------------------------------------------
DmCRY_NP_732407.1                   --------------------------------------------------
TrCRY-dash_ENSTRUT00000044961       --------------------------------------------------
zCRY-dash_NP_991249.1               --------------------------------------------------
TrCRY6a                             LNERDFTASVMHEGAQRRERMESDQRRLEGLPRGPAPRGRVRRTPTAKDR 794
AtCry1_AEE82696.1                   VPRNMVNTNQAQQRRAEPASNQVTAMIPEFNIRIVAESTEDSTAESSSSG 628
AtCry2_NP_171935.1                  --RDMKKS------------------------RGFDERELFSTAESSSSS 574
XlCPDphotolyase_NP_001089127.1      --------------------------------------------------
DmCPDphotolyase_NP_523653.2         --------------------------------------------------


hCRY1_NP_004066.1                   -------PKVQRQSTN---------------------------------- 586
cCRY1_NP_989576.1                   -------PKVQRQSTN---------------------------------- 621
XtCRY1_NP_001017311.2               -------PKVQRQSSH---------------------------------- 618
TrCRY1a_XP_011611545.1              -------SKVQRQSNH---------------------------------- 622
zCRY1a_CAH69047.1                   -------SKVQRQCSS---------------------------------- 619
zCRY1b_NP_571865.4                  -------SKIQRNDCN---------------------------------- 606
TrCRY2a_ENSTRUT00000004295          DGVACSLNKLQRQSTEV--------------------------------- 655
zCRY2a_BAA96848.1                   ESLSTS-HKLQRQIAEVTSVYASSGNQSSMRS------------------ 655
zCRY2b_NP_571867.2                  NGPSSSALKAQQQDNEKRREDAEGVAFST--------------------- 639
hCRY2_AAH35161.1                    --------------------------------------------------
cCRY2_NP_989575.1                   --------------------------------------------------
XtCRY2_BAI82612.1                   --------------------------------------------------
TrCRY3_ENSTRUG00000015322           --------------------------------------------------
zCRY3_BAA96850.1                    --------------------------------------------------
Tr[6-4]photolyase_XP_011607950      --------------------------------------------------
z[6-4]photolyase_BAA96852.1         --------------------------------------------------
cCRY4_NP_001034685.1                --------------------------------------------------
XtCRY4_AB534559                     --------------------------------------------------
zCRY4_AAI64413.1                    --------------------------------------------------
DmCRY_NP_732407.1                   --------------------------------------------------
TrCRY-dash_ENSTRUT00000044961       --------------------------------------------------
zCRY-dash_NP_991249.1               --------------------------------------------------
TrCRY6a                             FSVVPGGAVASLR------------------------------------- 807
AtCry1_AEE82696.1                   RRERSGGIVPEWSPGYSEQFPSEENGIGGGSTTSSYLQNHHEILNWRRLS 678
AtCry2_NP_171935.1                  ---------SVFFVSQSCSLASEGKNLEGIQDSSDQITTSLGKNGCK--- 612
XlCPDphotolyase_NP_001089127.1      --------------------------------------------------
DmCPDphotolyase_NP_523653.2         --------------------------------------------------


hCRY1_NP_004066.1                   ---
cCRY1_NP_989576.1                   ---
XtCRY1_NP_001017311.2               ---
TrCRY1a_XP_011611545.1              ---
zCRY1a_CAH69047.1                   ---
zCRY1b_NP_571865.4                  ---
TrCRY2a_ENSTRUT00000004295          ---
zCRY2a_BAA96848.1                   ---
zCRY2b_NP_571867.2                  ---
hCRY2_AAH35161.1                    ---
cCRY2_NP_989575.1                   ---
XtCRY2_BAI82612.1                   ---
TrCRY3_ENSTRUG00000015322           ---
zCRY3_BAA96850.1                    ---
Tr[6-4]photolyase_XP_011607950      ---
z[6-4]photolyase_BAA96852.1         ---
cCRY4_NP_001034685.1                ---
XtCRY4_AB534559                     ---
zCRY4_AAI64413.1                    ---
DmCRY_NP_732407.1                   ---
TrCRY-dash_ENSTRUT00000044961       ---
zCRY-dash_NP_991249.1               ---
TrCRY6a                             ---
AtCry1_AEE82696.1                   QTG 681
AtCry2_NP_171935.1                  ---
XlCPDphotolyase_NP_001089127.1      ---
DmCPDphotolyase_NP_523653.2         ---
```

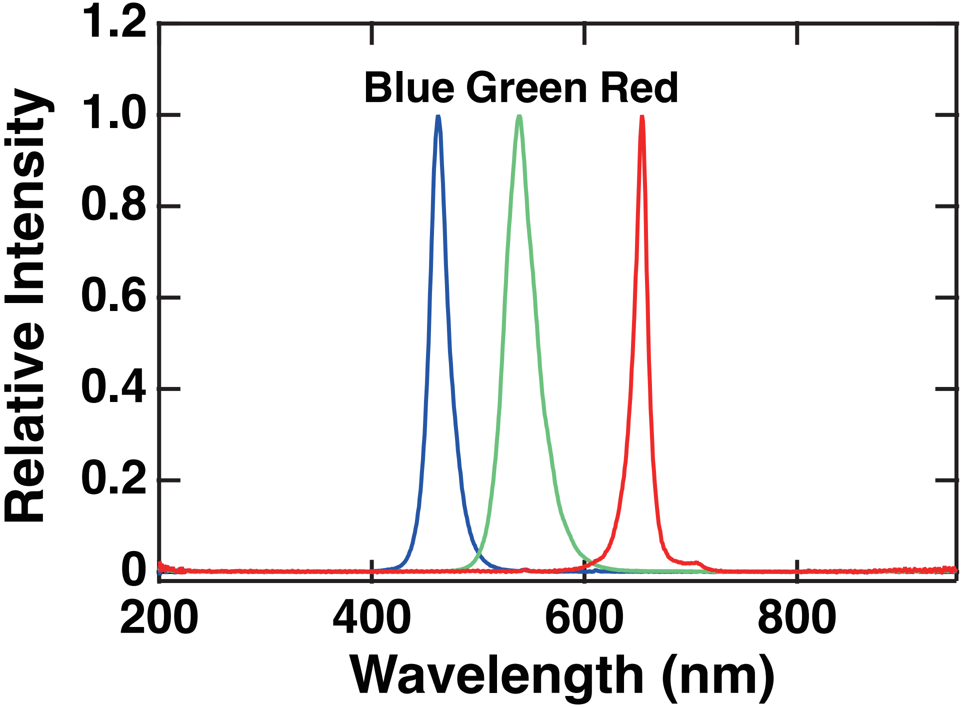

Figure S2 Emission spectra of LEDs used in this study.

LEDs used are Philips Lumileds LXK2-PR14-Q00 (λ_{max} = 462 nm; $\lambda_{1/2}$ = 20 nm) for blue light, Philips Lumileds LXK2-PM14-U00 (λ_{max} = 539 nm; $\lambda_{1/2}$ = 33 nm) for green light, and Epistar 33R-Y1-1 (λ_{max} = 654 nm; $\lambda_{1/2}$ = 15 nm) for red light. The spectra were measured using a photonic multichannel spectral analyzer (Hamamatsu Photonics, Model PMA-11; type C7473-36).
